# Tau seeds occur before earliest Alzheimer’s changes and are prevalent across neurodegenerative diseases

**DOI:** 10.1007/s00401-023-02574-0

**Published:** 2023-05-08

**Authors:** Matteo Manca, Heidi G. Standke, Danielle F. Browne, Mikayla L. Huntley, Olivia R. Thomas, Christina D. Orrú, Andrew G. Hughson, Yongya Kim, Jing Zhang, Curtis Tatsuoka, Xiongwei Zhu, Annie Hiniker, David G. Coughlin, Douglas Galasko, Allison Kraus

**Affiliations:** 1 Department of Pathology, Case Western Reserve University School of Medicine, 2103 Cornell Road, Cleveland, OH 44106 USA; 2 Laboratory of Persistent Viral Diseases, Rocky Mountain Laboratories, National Institute of Allergy and Infectious Diseases, National Institutes of Health, Hamilton, MT 59840 USA; 3 Department of Neurosciences, University of California San Diego, San Diego, CA 92093-0612 USA; 4 Department of Population and Quantitative Health Sciences, Case Western Reserve University School of Medicine, Cleveland, OH 44106 USA; 5 Department of Medicine, University of Pittsburgh, Pittsburgh, PA 15232 USA; 6 Department of Pathology, University of California San Diego, San Diego, CA 92093-0612 USA

**Keywords:** Tau, Protein seeds, RT-QuIC, Alzheimer’s disease, Alpha-synuclein, Tauopathy, Synucleinopathy

## Abstract

Tau neurofibrillary tangles are a hallmark of Alzheimer’s disease neuropathological change. However, it remains largely unclear how distinctive Alzheimer’s disease tau seeds (i.e. 3R/4R) correlate with histological indicators of tau accumulation. Furthermore, AD tau co-pathology is thought to influence features and progression of other neurodegenerative diseases including Lewy body disease; yet measurements of different types of tau seeds in the setting of such diseases is an unmet need. Here, we use tau real-time quaking-induced conversion (RT-QuIC) assays to selectively quantitate 3R/4R tau seeds in the frontal lobe which accumulates histologically identifiable tau pathology at late disease stages of AD neuropathologic change. Seed quantitation across a spectrum of neurodegenerative disease cases and controls indicated tau seeding activity can be detected well before accompanying histopathological indication of tau deposits, and even prior to the earliest evidence of Alzheimer’s-related tau accumulation anywhere in the brain. In later stages of AD, 3R/4R tau RT-QuIC measures correlated with immunohistochemical tau burden. In addition, Alzheimer’s tau seeds occur in the vast majority of cases evaluated here inclusive of primary synucleinopathies, frontotemporal lobar degeneration and even controls albeit at multi-log lower levels than Alzheimer’s cases. α-synuclein seeding activity confirmed synucleinopathy cases and further indicated the co-occurrence of α-synuclein seeds in some Alzheimer’s disease and primary tauopathy cases. Our analysis indicates that 3R/4R tau seeds in the mid-frontal lobe correlate with the overall Braak stage and Alzheimer’s disease neuropathologic change, supporting the quantitative predictive value of tau RT-QuIC assays. Our data also indicate 3R/4R tau seeds are elevated in females compared to males at high (≥ IV) Braak stages. This study suggests 3R/4R tau seeds are widespread even prior to the earliest stages of Alzheimer’s disease changes, including in normal, and even young individuals, with prevalence across multiple neurodegenerative diseases to further define disease subtypes.

**Supplementary Information:**

The online version contains supplementary material available at 10.1007/s00401-023-02574-0.

## Introduction

Self-propagating misfolded tau seeds are implicated as causal in tau-related disease processes. Towards this, use of sensitive techniques that specifically measure tau seeds are important to assess where and when seeds occur as part of disease-specific pathological sequelae. It is unclear if seed-competent tau is always synonymous with pathological tau, and how the observed histological staging of tau aggregates in Alzheimer’s disease (AD) corresponds with spread of pathologic tau seeds. Further, it is unknown if and how tau seeds spread in comorbid pathologies such as synucleinopathies with concurrent tau pathology. As tau progression correlates with neurodegeneration and cognitive deficits [3, 47], there is expanding interest in how tau seeding and propagation throughout the human brain correlates with the clinicopathology of different tauopathies and if it contributes to variability in clinical symptom presentation. While reports indicate tau seeding can be detected before significant tau neuropathology [12, 16, 26, 28], it is not established to what extent tau seeds broadly precede measures of histopathology and if this occurs equally for different disease-specific tau conformers [53]. In this study, we use selective and ultrasensitive diagnostic biomarker assays, known as real-time quaking-induced conversion (RT-QuIC) assays to directly and selectively measure AD (i.e. 3R/4R) tau seeds [31, 43] in AD and Lewy body disease (LBD) cases, as well as those representing different stages of AD pathology. Tau RT-QuIC exploits the seeded polymerization growth mechanism of tau filaments, with sensitivities in the low femtomolar range, allowing quantitation of disease-associated aggregates with unprecedented sensitivity and selectivity. Importantly, and in comparison to other seed amplification assays available, RT-QuIC seed amplification assays have more than a billion-fold dynamic detection range, and remarkable selectivity to distinguish among different disease-specific tau and α-syn seed conformers.

Tau occurs as 6 isoforms, with 0, 1, or 2 N-terminal inserts and 3 or 4 repeats (R) within the microtubule binding region. Tauopathies are characterized by the accumulation of 3R, 4R, or 3R/4R tau isoforms with AD being characterized by 3R/4R tau filament accumulation. Isoform and structural selectivity of specific RT-QuIC assays can discriminate seeding activity of 3R/4R tau filaments [31, 43] from conformationally distinct tau seeds of 4R [52] and 3R [51] tauopathies. RT-QuIC selectivity is dependent on the recombinant protein substrate used, with AD detection relying on substrates encompassing the amino acid sequence found in the AD tau filament core structure (i.e. τ306-378 [31] or K12 [43]) and 4R tauopathy detection using 4R tau substrate (i.e. K18 [52]). Other seed amplification assays using biosensor cells expressing fluorophore-tagged tau constructs to monitor aggregation have been used to provide important initial evidence that tau seeds occur early in AD disease processes in the brain [28], and progressively accumulate with Braak stages of disease [16], with normalized seeding activities suggested to be correlative with longevity of AD cases [1]. Additionally, biosensor cells expressing 3R, 4R or both 3R and 4R tau constructs have also indicated tau-based disease selectivity of cell infection with 4R tauopathies infecting only those cells expressing the 4R construct, 3R tauopathies infecting only 3R-expressing cells, and AD and CTE cases only initiating aggregation with the expression of both 3R and 4R tau or alternatively, overexpression of the 4R construct [63]. However, while indicative that substrate requirements can be seed specific, cells expressing both 3R and 4R or with overexpression of the 4R isoform would detect both 3R/4R and 4R tau seeding activities within a sample, and therefore are not necessarily selective for distinct and disease-specific seed conformers to distinguish mixed populations of 3R/4R and 4R tau seeds. As such, it remains unclear which conformers thereof and to what extent tau seeds can truly precede pathological processes. Our application of RT-QuIC methodologies to selectively quantitate AD specific (3R/4R) tau seeds in this study allows targeted assessment of the spatiotemporal aspects of seeds as it relates to neuropathologically defined and disease-associated processes.

Additionally, tau pathologies occur commonly in other types of neurodegenerative diseases, including primary synucleinopathies [27, 48]. Several studies have indicated that the co-occurrence of tau and α-synuclein (α-syn) aggregates can accelerate tau seeding processes in cellular and animal models [18, 23, 62]. While suggestive that α-syn and tau seeding processes might not be wholly independent, these studies often rely on transgenic mouse models expressing mutant forms of tau with indirect measures of insoluble tau accumulation [62] or histological readouts to assess aggregate accumulation [38]. As such, they do not directly evaluate the impact of multiple misfolded protein co-pathologies on the seed-competent conformers. Further, they may not have the sensitivity to detect low amounts of seeds, sub-fibrillar conformers, or structural polymorphs not visible when using immunohistochemistry.

Here, we use RT-QuIC assays to directly measure 3R/4R tau, 4R tau, and α-syn seeds in brain tissue from a spectrum of neurodegenerative diseases inclusive of AD, synucleinopathies, 4R tauopathies, and control cases. Our evidence suggests that 3R/4R tau seeds can be detected even in aged cases deemed clinically and neuropathologically “normal” and distal from the regions of the brain implicated in the very earliest stages of Alzheimer’s disease. 3R/4R tau seeds are also detectable and with prevalence in select younger cases. Seeding doses evaluated in the mid-frontal lobe predict overall levels of neuropathologic change in the brain even when tau neurofibrillary tangles (NFTs) are histologically visualized only in the transentorhinal cortex (i.e. Braak I). The billion-fold dynamic working range of RT-QuIC indicates that tau seeding activities that occur at Braak stage IV or above are higher on average in female versus male cases. Further, tau seeding doses differ with Lewy body stage in synucleinopathies, suggesting that 3R/4R tau seeds may also be useful to indicate stage of synucleinopathy. Our results, generated using ultrasensitive methods to detect disease-specific tau seeds, demonstrate that substantial quantities of 3R/4R tau seeds (i.e. 10,000–1,000,000 seeding doses) occur widely and in brain regions thought to be pathologically unaffected and can even be detected in neuropathologically normal brains.

## Methods

### Neuropathologic examination

Expert neuropathologists (L.A.H. and A. H.) applied current diagnostic criteria to cases to assign Thal phases [57], Braak tau stages [7], CERAD neuritic plaque stages [44], degree of AD neuropathological change, α-synuclein pathology [45], the presence of TDP-43 [37, 46], and aging-related tau astrogliopathy (ARTAG) co-pathology [29] at the time of autopsy. Final neuropathology diagnosis for each case was rendered using standard semi-quantitative assessments for each pathology in each brain region [14, 30, 37, 41, 45, 59]. All procedures were performed with prior informed consent in accordance with UCSD Institutional Review Board guidelines.

Sixty-seven cases from UCSD were assessed as shown in the main manuscript (Supplementary Table 1, online resource), including subjects with neuropathologically confirmed AD (high *n* = 15; intermediate *n* = 1), Lewy body disease with subgroups of clinically indicated Parkinson’s disease (*n* = 8) and dementia with Lewy bodies (*n* = 13), multiple system atrophy (*n* = 6), progressive supranuclear palsy (*n* = 6), corticobasal degeneration (*n* = 6), and controls (Braak ≤ II, inclusive of *n* = 2 young Huntington’s cases, and *n* = 10 normal or mild AD changes).

Frozen cortex samples of three additional younger cases (designated controls 10–12, Supplementary Fig. 2, online resource) and an additional AD case (Fig. 5e, Supplementary Fig. 4, online resource) were obtained from the brain bank at Case Western Reserve University and University Hospitals of Cleveland, under an approved Institutional Review Board protocol.

### Digital histology

Six μm sections from formalin fixed paraffin embedded sections from middle frontal cortex (MFC) were immunostained for phospho-tau and AD-specific tau conformers (AT8, Thermofisher MN1020, 1:500, formic acid antigen retrieval, GT38 [19, 20] Abcam ab246808, 1:500, citrate heat mediated antigen retrieval). Briefly, slide sections were deparaffinized and rehydrated using graded ethanols, after which the appropriate antigen retrieval method described above was implemented. Slides then were placed in a 30% H_2_O_2_ in methanol solution for 30 min, washed in 0.1 M *tris*(hydroxymethyl)aminomethane (TRIS) at pH 7.6, blocked with 2% fetal bovine serum (FBS) in 0.1 M TRIS, and then slides were incubated in primary antibody at 4 °C overnight. The second day, after washing with 1% TRIS solution and blocking with 2% FBS, slides were incubated in biotinylated horse anti-mouse IgG secondary antibody at 1:1000 concentration (Vector laboratories, Burlingame California) for one hour at room temperature, then for an additional one hour in avidin/biotin-based peroxidase (Vector laboratories, Burlingame California). The chromogen used was 3,3’-diaminobenzidine (DAB: Vector laboratories, Burlingame California), developed for seven minutes and counterstained with hematoxylin. Slides were then dehydrated in graded ethanols, treated with xylene, and coverslipped. Whole slide images of histology slides at 20 × magnification were obtained using a Zeiss AxioScan Z1 (Oberkochen Germany, czi file type). Pixel size of 6.5 μm^2^ (i.e., pixel resolution of 0.325 μm), camera resolution of 2560 × 2160, and a bit depth of 16.

Digital measurements of pathological burdens were derived using the open-source program QuPath (0.2.0 m^2^ Belfast, Northern Ireland [5]) which calculated percent area occupied (%AO) for pathological tau accumulations. Grey and white matter regions of interest selected by a single trained rater (DGC) were evaluated for all cases using previously validated sampling methods to determine % area occupied (%AO) [9, 10, 17]. Briefly, for GM annotations, a modified belt-transect method was used to select the longest region of representative parallel-oriented cortex using parallel lines drawn at the pial surface and grey-white junction to generate a rectangular region of interest. For WM sampling, a rectangular area of deep white matter, away from U-fiber tracts was selected as regions of interest. Next random sampling was applied to each GM and WM ROI using 175 μm^2^ tiles with 70% dropout to reduce sampling bias. Color deconvolution intensity thresholds were optimized per each staining run at each site by averaging values of red-blue-green color vectors and optimal minimal optical density values visually tuned from five representative slides per staining run (see Supplementary methods, online resource). Downsample value of 2 with a gaussian sigma value of 1 was employed for detections.

### Protein expression and purification

#### K23Q recombinant α-Syn purification

K23Q α-syn recombinant protein was purified as per Groveman et al*.* [22]. Briefly, 5 ml of LB medium with 50 μg/mL kanamycin was inoculated from a glycerol stock of BL21(DE3) E. coli bacteria with pET28 plasmid encoding K23Q α-syn protein (accession No. NM_000345.3) amino acid residues 1–140. Following a ~ 5-h incubation (225 rpm agitation, 37 °C), the starter culture was added to 1 L of the auto-induction medium with 50 μg/mL kanamycin. Cells were grown at 37 °C, 225 rpm, overnight and harvested the next day by centrifuging at 3273 × g, 4 °C, 10 min. The pellet was gently resuspended in 10% volume of room temperature osmotic shock buffer, (25 mL per 250 mL of cell culture before centrifugation) and incubated at RT for 10 min. Following a spin at 9000 × g, 20 °C, 20 min, each pellet was gently resuspended in 10 mL of ice-cold water and the suspensions were pooled into 20 mL per tube. Next, 20 μL of saturated MgCl_2_ was added to each suspension, followed by incubation on ice with mild rocking for 3 min. After a centrifugation at 9000 × g, 4 °C, 30 min, the supernatant was collected in a glass beaker and subjected to rapid continuous stirring. pH was adjusted to 3.5 followed by gentle stirring at room temperature for 10 min. Tubes were centrifuged at 9000 × g, 4 °C, 30 min, and the supernatant was collected in a fresh beaker with continuous agitation with a stir bar, and pH adjusted to 7.5. After a 0.45 μm filtration, the protein extract was loaded onto a 5 mL Ni–NTA column (Qiagen) on an Äkta Pure chromatography system (GE). After a wash with 20 mM Tris, pH 7.5 and a wash in 50 mM imidazole, 20 mM Tris, pH 7.5 an initial peak was observed, which was not collected. Following a linear gradient up to 500 mM imidazole in 20 mM Tris, pH 7.5, another peak was collected between 150 and 375 mM imidazole. This peak was loaded onto a Q-HP column (GE) and washed with 20 mM Tris, pH 7.5. An additional 100 mM NaCl, 20 mM Tris, pH 7.5 wash was done, then a linear gradient up to 500 mM NaCl in 20 mM Tris pH 7.5 was performed and a peak was recovered between 300 and 350 mM NaCl. After a 0.22 μm filtration, the protein was dialyzed against water overnight at 4 °C using a 3 kDa MWCO dialysis membrane. Protein concentration was determined with a UV–VIS spectrophotometer using a theoretical extinction coefficient at 280 nm of 0.36 (mg/mL) ^−^1 cm ^−^1. Then the protein was lyophilized in aliquots and stored at − 80 °C for a final concentration of ~ 1.0 mg/ml once resuspended in 40 mM phosphate buffer (pH 8.0).

#### Tau substrate purification

Tau recombinant proteins were purified as per Kraus et al. and Metrick et al. [31, 43]. Briefly, BL21(DE3) E. coli cells engineered as previously described to express cysteine-free K12 tau fragment with a histidine tag (K12CFh), cysteine-free K18 tau fragment with a histidine tag (K18CFh), cysteine-free K19 tau fragment with a histidine tag (K19CFh), or τ306 (inclusive of amino acid residues 306–378 with a cysteine to serine point mutation at residue 322) were induced with the Overnight Express Autoinduction System [56]. Bacterial pellets were recovered after centrifugation at 3273xg for 35 min at 4 °C and resuspended in Buffer A (10 mM Tris, pH 8.0, 500 mM NaCl, and 5 mM imidazole). Cells were lysed with four cycles of sonication (Qsonica Q700, 45 s each, ~ 95 watts, 1-min pause between each cycle) and centrifuged at 9685xg for 1 h at 4 °C. The supernatant was recovered, filtered with a 0.45-μm syringe filter and run through a His-Trap FF column (GE Healthcare 17-5255-01) for nickel affinity chromatography. The column was washed with 10 column volumes (CV) of Buffer A and subsequently with 13 and 21% of Buffer B (10 mM Tris, 500 mM NaCl, 200 mM imidazole, pH 8.0) over 5 and 7 CV, respectively. Protein was eluted over a linear gradient of 23–100% Buffer B (i.e. 46–200 mM imidazole) over 8 CV. 2 mM dithiothreitol (DTT) was added to the fractions collected and fractions were analyzed by SDS-PAGE, pooled according to purity, mixed with 4 volumes of acetone, and precipitated overnight at 4 °C. A 20-min centrifugation at 12,439xg at 4 °C was used to pellet protein. The supernatant was discarded and pellets washed with the same volume of acetone/2 mM DTT. After another centrifugation at the same conditions, the supernatant was decanted and pellets solubilized in 8 M guanidine-HCl in PBS. Protein was desalted into PBS pH 7.4 using PD-10 desalting columns (Cytiva, 17-0851-01) and the final protein concentration was adjusted to ~ 0.75 mg/mL and aliquots stored at − 80 °C until use. Reproducible performance of independent protein batches was verified over at least two (AD RT-QuIC) and more than nine (K12 and 4R RT-QuIC) independent protein purifications.

### Tissue homogenization

Frozen tissue was obtained, with the contralateral half being fixed and used for histopathology as described above. Multiple brain sections were dissected from the samples of mid-frontal lobe tissue to ensure representative samplings. 10% w/v brain tissue homogenates were prepared in ice-cold 1 × PBS with cOmplete protease inhibitors, EDTA-free (Roche) and homogenized using 1 mm zirconia/silica beads (BioSpec Products) in a BeadMill (Fisherbrand). Homogenates were placed on ice for 5 min before centrifugation at 2000×*g* for 2 min. Supernatant was collected, aliquoted and stored at − 80 °C.

### Proteinase K digestion

10% w/v brain homogenate was mixed with 1% sarkosyl and 50 µg/ml Proteinase K (PK) (final concentration) and incubated at 37 °C with shaking for 1 h. Pefabloc was added to the samples before subsequent comparative end-point dilution analysis of PK-treated versus untreated samples. PK digestion efficacy was confirmed using SDS-PAGE analysis of the samples.

### Mass spectrometry analysis

The equivalent of 10 µg of total protein was mixed with 5 µl of 50 mM DTT and incubated at 37 °C for 30 min. A volume of 5 µl of 100 mM Iodoacetamide was added and well mixed before incubating samples in the dark for 30 min at RT. Volume was brought to 45 µl with 50 mM Tris pH 8 and digested overnight at 37 °C with 5 µl of 0.1 µg/µl Trypsin. Samples were spun to recover condensation. For reverse phase LC–MS/MS analysis, three hundred nanograms of total protein were analyzed by LC–MS/MS using a Orbitrap Exploris 480 Mass Spectrometer (Thermo Scientific, San Jose, CA) equipped with a nanoAcquity^™^ Ultra-high pressure liquid chromatography system (Waters, Taunton, MA). Mobile phases were organic phase A (0.1% formic acid in water) and aqueous phase B (0.1% formic acid in acetonitrile). Peptides were loaded onto a nanoACQUITY UPLC^®^ 2G-V/M C18 desalting trap column (180 μm × 20 mm nano column, 5 μm, 100 Å) at flow rate of 0.300 µl/minute. Subsequently, peptides were resolved in a nanoACQUITY UPLC^®^ BEH300 C18 reversed phase column (75 μm × 250 mm nano column, 1.7 μm, 100 Å; Waters, Milford, MA) followed by a gradient elution of 1–95% of phase B over 150 min (isocratic at 1% B, 0–1 min; 2–40% B, 2–120 min; 95% B, 121-131 min; and 1% B, 131–150 min). A nano ES ion source, 1.5 kV spray voltage, and 270 °C capillary temperature was utilized to ionize peptides. Full scan MS spectra (m/z 380–1800) were acquired at a resolution of 120,000 followed by twenty data dependent MS/MS scans. MS/MS spectra were generated by collision induced dissociation of the peptide ions (normalized collision energy = 35%) to generate a series of b-and y-ions as major fragments.  LC–MS/MS raw data were then acquired using the Xcalibur software (Thermo Fisher Scientific, version 2.2 SP1). Raw data were processed using Raw Converter (Scripps Research Institute version 1.2.0.1) The peak lists (mgf) files were searched by Mascot (version 2. 7, Matrix Science London, UK) against the Uniprot dataset. Mascot search settings were as follows: trypsin enzyme specificity; mass accuracy window for precursor ion, 10 ppm; mass accuracy window for fragment ions, 0.6 Da; carbamidomethylation of cysteines as fixed modifications; oxidation of methionine as variable modification; and one missed cleavage. Peptide identification criteria were a mass accuracy of ≤ 10 ppm, an expectation value of *p* < 0.05, and an estimated False Discovery Rate (FDR) of less than 2%.

### Real-time quaking-induced conversion (RT-QuIC) analysis

K12 [43], AD [31], 4R [52] and α-syn [22] RT-QuIC assays were performed as described below. Tau RT-QuIC assays use mouse tau knock-out brain homogenate, and as a negative control as we have previously described [31, 43].

Tau RT-QuIC assays to quantify 4R and 3R/4R tau seeds were performed based on previously published protocols [31, 43, 52]. Assay specific tau recombinant protein substrates were purified as described above. Tau mouse knock-out brain tissue was derived from breeding colonies of B6.Cg-Mapt^tm1(EGFP)Klt^ Tg(MAPT)8cPdav/J (#005491, Jackson) with genotype selection for mouse tau knock-out mice without the human tau transgene (NIH RML Animal Care and Use Committee approved protocol RML 2019-043). Endpoint-dilution analysis was conducted with dilution of homogenates in diluent buffer (0.526% mouse tau KO brain homogenate/N2/10 mM Hepes pH 7.4) with dilutions used to seed 4R RT-QuIC reactions [7.5 μM K18CFh and 3.75 μM K19CFh recombinant tau, 90 μM poly-glutamate (Sigma, P1818), 40 mM HEPES pH 7.4, 200 mM sodium citrate, and 10 μM thioflavin T (ThT) with one zirconia/silica bead), K12 RT-QuIC reactions (6.5 μM K12CFh, 40 μM heparin, 40 mM HEPES, 400 mM NaF, and 10 μM ThT), or AD RT-QuIC reactions (12 μM of recombinant tau substrate (τ306 and K19CFh at a 1:3 molar ratio), 40 μM heparin, 10 mM HEPES pH 7.4, 400 mM sodium chloride, and 10 μM thioflavin T (ThT) with one silica bead] in a 384-well optical plate (Thermo Scientific Nunc, 242764). Plates were sealed (Nunc 232702) and incubated at 42 °C (for K12 RT-QuIC), 37 °C (for AD RT-QuIC), or 30 °C (for 4R RT-QuIC) with alternate 1-min cycles of orbital shaking (500 rpm) and rest in a BMG FLUOstar Omega plate reader. Fluorescence reads were taken every ~ 45 min, bottom read, using 450–10 nm excitation and 480–10 nm emission.

α-syn RT-QuIC rapid (RT-QuICR) assays were performed in clear bottom black 96- or 384-well plates (Nalgene Nunc International). Individual wells were preloaded with 2–6 glass beads (0.8 mm in diameter, OPS Diagnostics). 1–2 µL of brain homogenate was used to seed RT-QuIC reaction mix of 40 mM sodium phosphate buffer, 170 mM NaCl, 0.1 mg/ml K23Q recombinant α-syn (filtered through a 100 kD MWCO filter prior to use), 10 μM thioflavin T (ThT). The plates were closed with a plate sealer film (Nalgene Nunc International) and incubated at 42 °C in a BMG FLUOstar Omega plate reader with cycles of 1 min shaking (400 rpm double orbital) and 1 min rest for at least 60 h. Fluorescence reads were taken as described above.

### Immunoprecipitation

1.875 mg of Dynabeads (Invitrogen 10007D) were separated from the storage buffer and resuspended in 200 µl of washing buffer (0.1 M NaCit/0.025% Tween 20, pH 5). 15 µg of either isotype control (mouse IgG1, Invitrogen 14-4714-85) or AT8 (Invitrogen MN1020) antibodies were added to the bead suspension and incubated at room temperature, with rotation, for 30 min. Supernatant was discarded, beads were rinsed once with 200 µl of washing buffer and transferred into a fresh tube with the same amount of washing buffer. 1.21 mM of cross-linking reagent (BS3 crosslinker proteochem c1103) was added to the bead suspension and incubated at room temperature, with rotation, for 45–60 min. Unreacted cross-linking reagent was quenched with 56.3 mM Tris–HCl at room temperature, with rotation, for 15 min. Supernatant was discarded and bead-Ab complexes were rinsed once with 200 µl of washing buffer by gently pipetting. Buffer was removed and 250 µl of brain homogenate (in 1X N2/0.05% Tween 20/PBS) were added and mixed gently with the bead-Ab complexes at room temperature, with rotation for 35 min. Supernatant was then recovered and used to make 1:10 serial dilutions in diluent buffer (0.526% BH/1X N2/10 mM Hepes pH 7.4). One microliter of such dilutions was used to seed K12 RT-QuIC reactions.

### Immunoblotting

Protein transfer to PVDF iBlot 2 Transfer Stack (Invitrogen, IB24002) was done using the Invitrogen iBlot 2 Dry Blotting System (Invitrogen, IB21001S) according to the P0 method in the user manual. Immunoblotting was conducted using the iBind Flex Western Device (Invitrogen, SLF2000), iBind Flex Card (Invitrogen, SLF1020), and iBind Flex Solutions (Invitrogen, SLF2020) according to the user manual. Primary (Rabbit IgG mAb Tau (D1M9X) XP^®^, Cell Signaling Technology, 46687S) and secondary (Rabbit whole IgG PAP pAb, Jackson ImmunoResearch Laboratories, 323-005-024) antibodies were diluted 1:1000 in wash buffer. The membrane was developed in AttoPhos^®^ Substrate (Promega, S1000) and imaged on an ImageQuant LAS4000mini (GE).

### Sarkosyl insoluble tau preparation

Preparation of sarkosyl-insoluble tau followed an adapted [21] protocol. A bead mill homogenizer was used to make 10% w/v brain homogenates using a homogenization buffer composed of 10 mM Tris–HCl (Sigma, T6066) pH 7.4, 0.8 M NaCl (Sigma, S7653), 1 mM EGTA (Sigma, 4100-OP), and 10% w/v D-sucrose (Fisher, 57-50-1) filter sterilized with 22 μM filter. Homogenates were centrifuged at 20,000xg for 20 min at 4 °C. Supernatant was collected and the pellet was resuspended in homogenization buffer and re-centrifuged at 20,000×*g* for 20 min at 4 °C. The supernatants were pooled and 10% v/v of 10% w/v n-lauryl sarcosine (Sigma, L5125) was added and the solutions were rotated at room temperature for 60 min. The solution was centrifuged at 100,000×*g* for 60 min at 4 °C and the resulting pellet was resuspended with 1X PBS and stored at − 80 °C.

### Estimation of sarkosyl-insoluble extract concentration

A standard curve of protein concentration was created using ImageJ and SDS page analysis. Prior to SDS page analysis, sarkosyl-insoluble extracts were sonicated using a cup-horn sonicator at 130 watts for 1 min. Known w/v concentrations of recombinant protein were plotted against the ImageJ peak area values in Excel to create a linear regression. The peak areas of the sarkosyl-insoluble extracts were used within the linear regression model to estimate the w/v concentration of the sarkosyl-insoluble extract. From the concentration obtained from the linear regression and the known volume of extract, the mass of sarkosyl-insoluble product recovered from the known mass of brain tissue used in the protocol was determined and assumed to be 100% (though the recovery of product was likely less efficient). This value was used to estimate the sarkosyl insoluble w/v concentration present in 10% w/v brain homogenate. The sarkosyl-insoluble extract concentration used in subsequent SDS page analysis was divided by the estimated sarkosyl-insoluble concentration of 10% brain homogenate to yield the ratio of brain tissue equivalence loaded.

### Determination of ThT fluorescence threshold for RT-QuIC positives

The mean and standard deviation of the negative control baseline across 4 replicates, excluding the first 9 reads to allow for temperature acclimatization and establishment of a consistent baseline, were used to calculate corresponding z-score values for each read. Replicate wells whose z-score value exceeded ± 1.96 (outside of the 95% confidence level) were excluded from threshold calculations. The threshold for a positive read was then calculated using the average of the remaining wells summed with 3 × standard deviation of the baseline for α-syn RT-QuICR and 100 × for tau RT-QuIC assays. Comparison of average baseline fluorescence reads of mouse tau KO brain homogenate and RT-QuIC negative human brain homogenate validated statistical comparability of baselines (*t* test, *p* = 0.35).

### Statistical analysis

Statistical analysis to compare mean seeding doses between neuropathological diagnosis was conducted as indicated using GraphPrism 9.1.2. Seeding doses are shown as log_10_ values. *p *values of seeding activities of each group compared to AD, or each group compared to baseline values determined with tau knock-out controls were determined using one-way ANOVA. Digital neuropathology % area occupied (%AO) data was transformed (square root) as previously reported [9, 10] and correlation analysis used to compute Pearson correlation coefficients. To investigate the relationship between sex and tau seeding doses, we used median quantile regression controlling for binary age (≤ 70 or ≥ 70), binary Braak stage grouped by severity (0–III or IV–VI), and the interaction between binary Braak stage and sex. Median quantile regressions were conducted in R (4.2.1).

### Spearman Kärber

Endpoint-dilution analysis was conducted where indicated and used to determine seeding doses as per methods of Spearman-Kärber [15] as has been previously described [31, 61].

### Data availability

Data is available upon request.

## Results

### 3R/4R tau seeds are detectable in AD, synucleinopathy, 4R tauopathy, and control cases

**Fig. 1:**
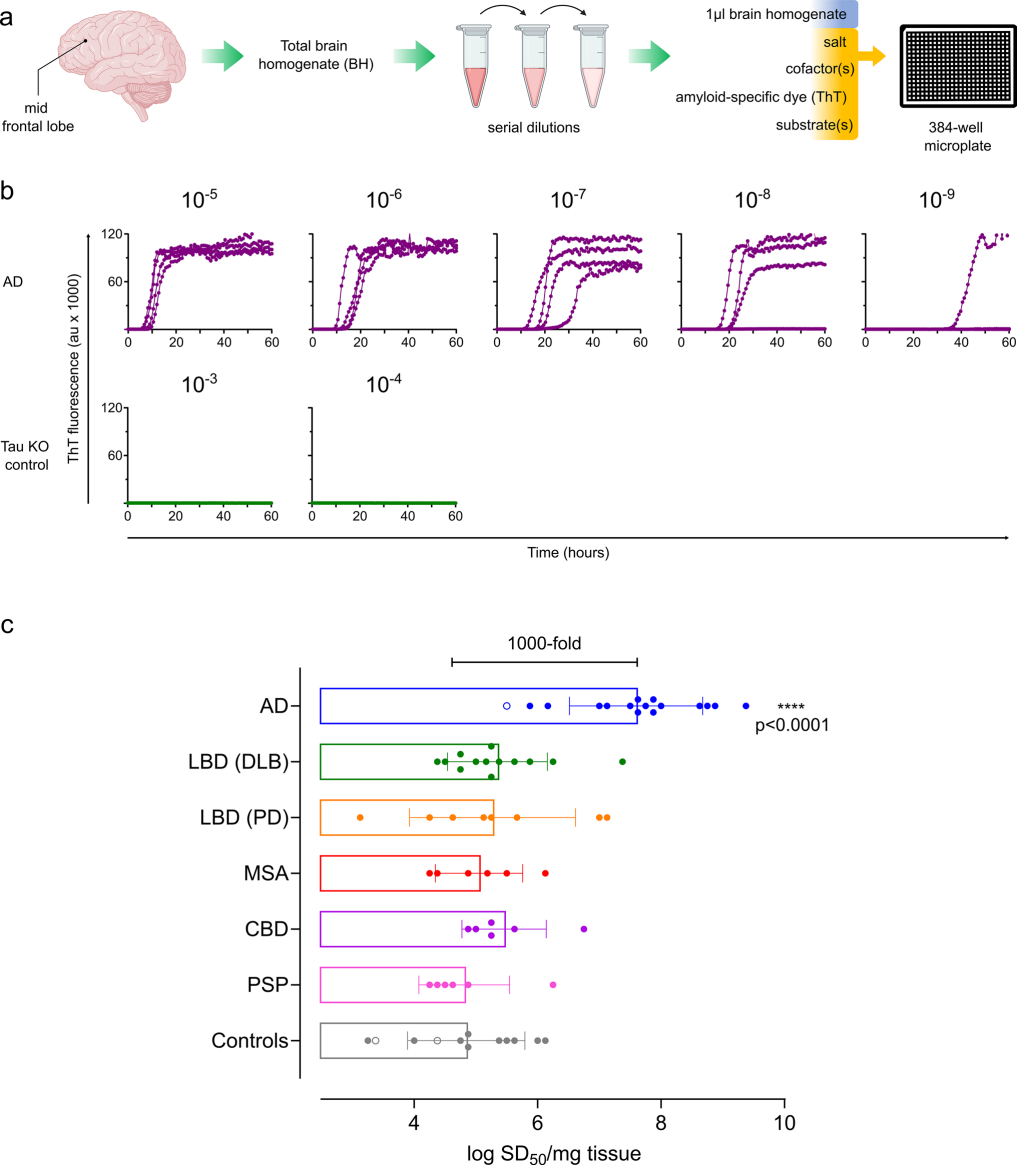
3R/4R tau seeds are detectable in all cases inclusive of AD, synucleinopathies, 4R tauopathy, and controls. **a** Endpoint-dilution analysis and subsequent analysis by real-time quaking-induced conversion (RT-QuIC) assay was used to determine 3R/4R tau seeding activities in total brain homogenates (BH) from the mid-frontal lobe. **b** RT-QuIC readouts from a representative Alzheimer’s disease (AD) case and a control of mouse tau knock-out (KO) BH are shown. Each curve shows the Thioflavin T (ThT) fluorescence units (arbitrary units) of an individual well seeded with the brain homogenate dilution listed on the top, with quadruplicate well analysis. Seeding activities occur with up to 10^–9^ brain tissue dilutions of AD whereas no seeding activity is noted in the tau KO control. **c** Each data point represents the average log_10_ seeding dose (SD_50_) of an individual case (*n* = 2–4 independent determinations) with the primary neuropathologic diagnosis as indicated. Bars indicate means of all cases ± standard deviation. Cases analyzed included AD (severe *n* = 15; intermediate *n* = 1 labeled as an open circle), Lewy body disease (LBD) inclusive of those cases with clinically indicated dementia with Lewy bodies (DLB) [LBD (DLB) (*n* = 13)] and Parkinson’s disease (PD) [LBD (PD), (*n* = 8)], multiple system atrophy (MSA) (*n* = 6), corticobasal degeneration (CBD) (*n* = 6), progressive supranuclear palsy (PSP) (*n* = 6), and controls, inclusive of Braak 0-II [*n* = 2 Huntington’s disease (open circles), normal or mild AD changes *n* = 10]. AD seeding doses are ~ 1000-fold higher on average than synucleinopathy, tauopathy, and control cases (*p* < 0.0001). All cases, including controls, have significant seeding activity when compared to the tau mouse knock-out negative control (*p* < 0.0001)

Tau RT-QuIC assays were used to quantitate 3R/4R tau seeds in mid-frontal lobe brain tissue (Fig. 1) from 67 cases including subjects with neuropathologically confirmed AD (*n* = 16), Lewy body disease (LBD: Parkinson’s disease (PD) *n* = 8, dementia with Lewy bodies (DLB) *n* = 13), multiple system atrophy (MSA) (*n* = 6), progressive supranuclear palsy (PSP) (*n* = 6), corticobasal degeneration (CBD) (*n* = 6), and controls inclusive of two Braak 0 cases with Huntington’s disease, and 10 cases with no or mild Alzheimer’s changes (Braak 0–II) (*n* = 12) (Supplementary Table 1, online resource). Seeding activities were assessed in total brain homogenates subjected only to a brief, low speed spin, to detect all seed-competent tau conformers regardless of their potential distribution to insoluble or soluble fractions.

3R/4R tau seeding activity was detectable in all cases (*p* < 0.0001 when compared to the tau KO mouse BH negative control), being on average 100-1000-fold higher in cases with a primary AD diagnosis compared to controls, 4R tauopathies, and synucleinopathies (Fig. 1c, Supplementary Fig. 1, online resource), consistent with our prior observations [31, 43]. Quantitative tau seeding activities (seeding doses, or SD_50_s) were the most variable between individual LBD (PD) cases spanning a range of ~ 4 logs (mean log SD_50_/mg = 5.27, range 3.13–7.13). Unexpectedly, up to 1-million-fold seeding doses were also observed in the controls inclusive of cases ≤ Braak II and those deemed neuropathologically normal, suggesting levels of 3R/4R tau seeds occur abundantly and as a frequent co-occurrence in both non-AD diseases and age-comparable controls.

### Widespread 3R/4R tau seeding activities prior to histologically identifiable pathological tau and overall AD neuropathologic change

#### 3R/4R tau seeds increase with overall Braak stage, while being detectable in Braak 0 cases

**Fig. 2:**
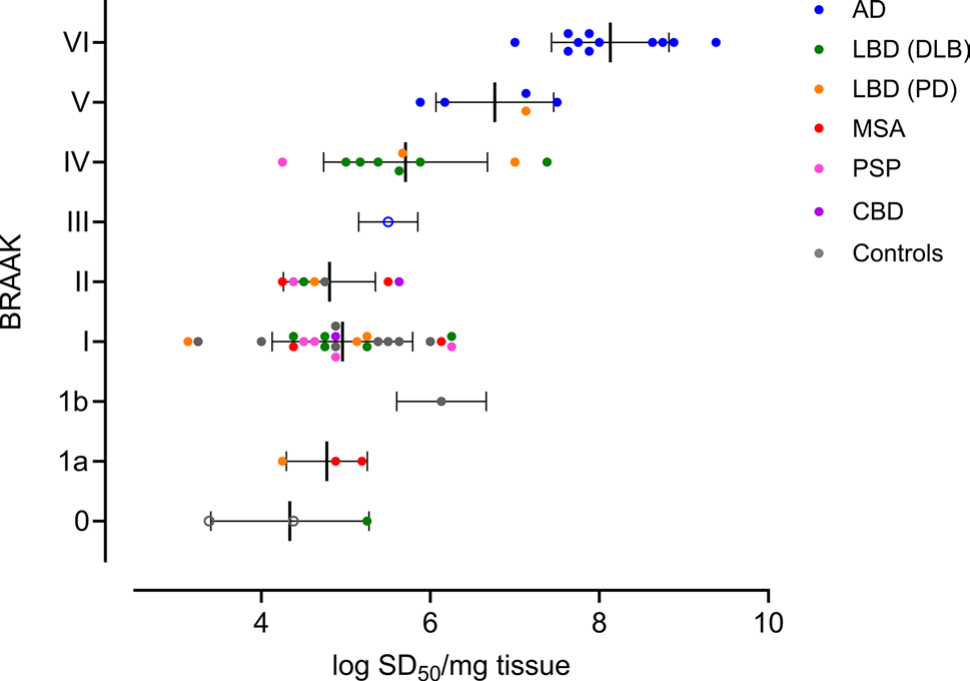
3R/4R tau seeds are detectable in the mid-frontal lobe at all Braak stages and increase with higher Braak stages. Seeding doses are shown (log SD_50_/mg) with the corresponding overall Braak stage of the case. Each data point indicates the mean SD_50_ of a case with standard deviation as indicated by the horizontal bars. AD (*n* = 16), LBD (PD) (*n* = 8), LBD (DLB) (*n* = 13), MSA (*n* = 6), CBD (*n* = 2), PSP (*n* = 6), and controls inclusive of those cases ≤ Braak II (*n* = 12)

3R/4R tau NFTs are thought to occur progressively in connected regions of the brain and are used to stage AD neuropathological change [7]. We determined if 3R/4R seeding activities are quantitatively related to Braak stage and NFT accumulation [6, 7]. Median quantile regression results indicated that high (≥ IV) versus low (≤ III) Braak stage as a histological indicator of regional tau accumulation is significantly related to tau seeding doses in the frontal cortex (*p* = 0.003) (Fig. 2). Tau seeds are detectable in the frontal lobe even in Braak 0-II cases being representative of stages at which the frontal lobe (and in cases of confirmed Braak 0, the entire brain) lacks histologically visible tau deposits. However, while seeding activities at Braak stage 0–III were comparatively lower overall than those observed at Braak V and VI, the comparative means of Braak stage 0, I, II, III, and even IV were not statistically different from each other (one-way ANOVA), at least with the case numbers evaluated here. This suggests that tau seeds are widespread and prevalent prior to any histological evidence of tau accumulation but increase most significantly with staged Braak pathology after Braak III/IV.

#### 3R/4R seeding doses correlate with digital neuropathological assessments of tau load

**Fig. 3:**
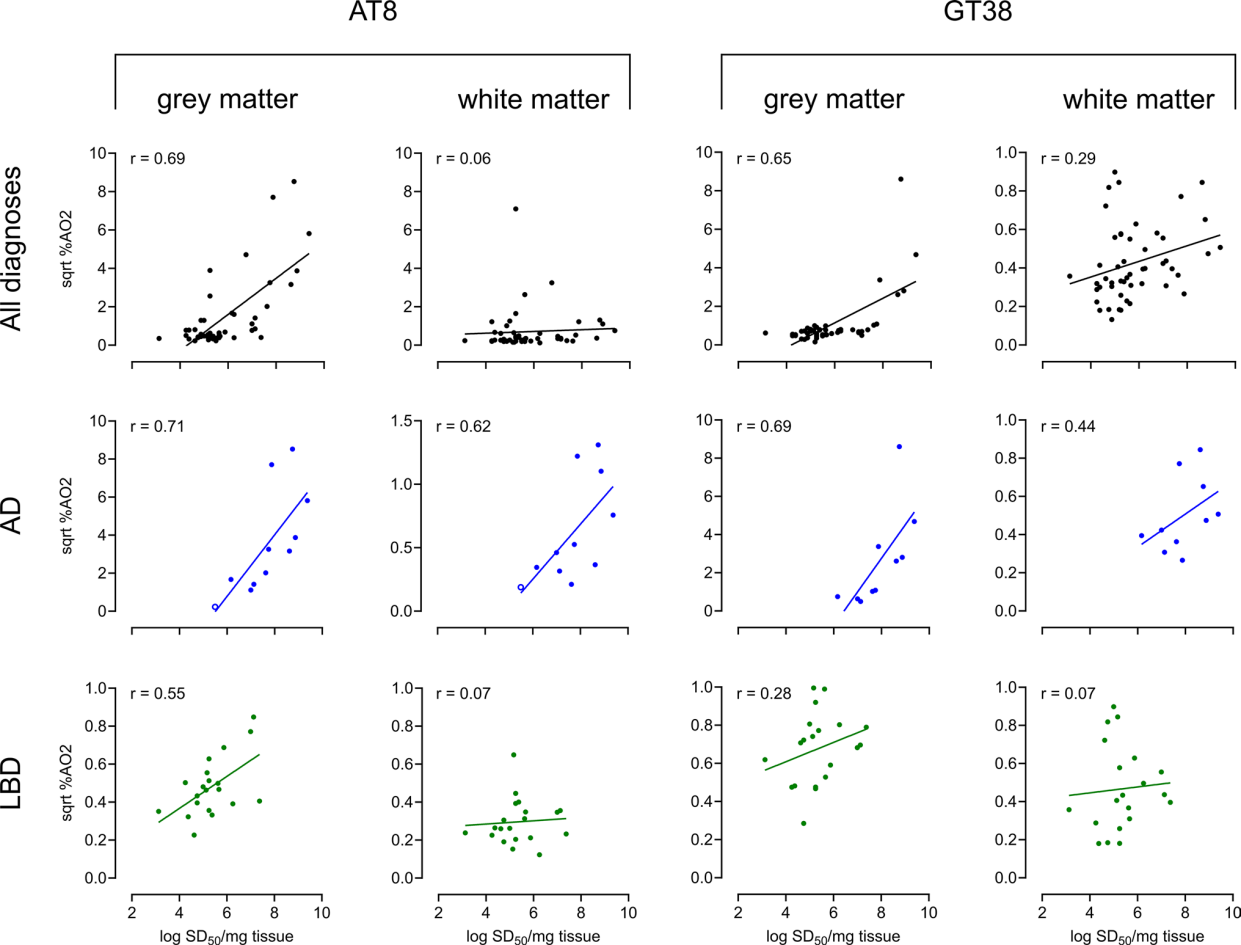
Seeding doses correlate with pathological tau load in AD and LBD cases. Digital neuropathology of frontal cortex sections that were immunostained with phospho-tau (AT8) or an AD-specific conformational antibody (GT38) was used to determine % area occupied (%AO) by tau in the grey (GM) and white matter (WM) [square root (sqrt) GM %AO2; sqrt WM %AO2]. The top panel shows AT8 and GT38-tau immunoreactive deposits as they correlate with seeding doses (log SD_50_/mg brain tissue) inclusive of all cases assessed, with subpanels for AD and LBD cases as indicated. Each data point indicates the mean log SD_50_ determined for an individual case. GT38 stained cases: AD = 10, LBD (DLB) = 12, LBD (PD) = 8, MSA = 5, CBD = 6, PSP = 5, Controls = 4. AT8 stained cases: AD = 11, LBD (DLB) = 12, LBD (PD) = 8, MSA = 4, CBD = 6, PSP = 5, Controls = 3

To assess how seeding doses correlated with histological detection of tau, we immunostained mid-frontal cortex sections with an AD-specific conformational monoclonal (GT38) [19] or phospho-tau (AT8) antibody and used digital neuropathology assessments [5] to quantitate pathological tau burden (Fig. 3). For this analysis, we excluded comparatively younger cases < 45 years (see Supplementary Table 1 and Supplementary Fig. 2, online resource). GT38% area occupied (%AO) from frontal lobe sections correlated with seeding doses detected across cases and in AD cases in grey matter (*r* = 0.69, *p* = 0.03) but not white matter (*r* = 0.44, *p* = 0.20) and did not correlate with seeding activities in LBD cases (*r *= 0.28, *p* = 0.20 in grey matter) (Fig. 3). Phospho-tau (AT8) specific staining was comparable to that of GT38 with seeding doses strongly correlated in AD cases to %AO AT8 and tau for both grey and white matter (*r* = 0.71, *p* = 0.02 and *r* = 0.62, *p* = 0.04 respectively) (Fig. 3). However, LBD cases showed a modest correlation of seeding doses with %AO in grey matter (*r* = 0.55, *p* = 0.01) and did not correlate with white matter %AO for tau. %AO in grey matter was also much lower for both GT38 and AT8 staining in LBD cases, even in cases with comparable seeding doses to those of AD cases, suggesting that seeding doses as it relates to histological tau load is not equivalent between AD and LBD cases.

#### 3R/4R seeding activities correspond with Aβ pathology and overall AD neuropathologic change

**Fig. 4:**
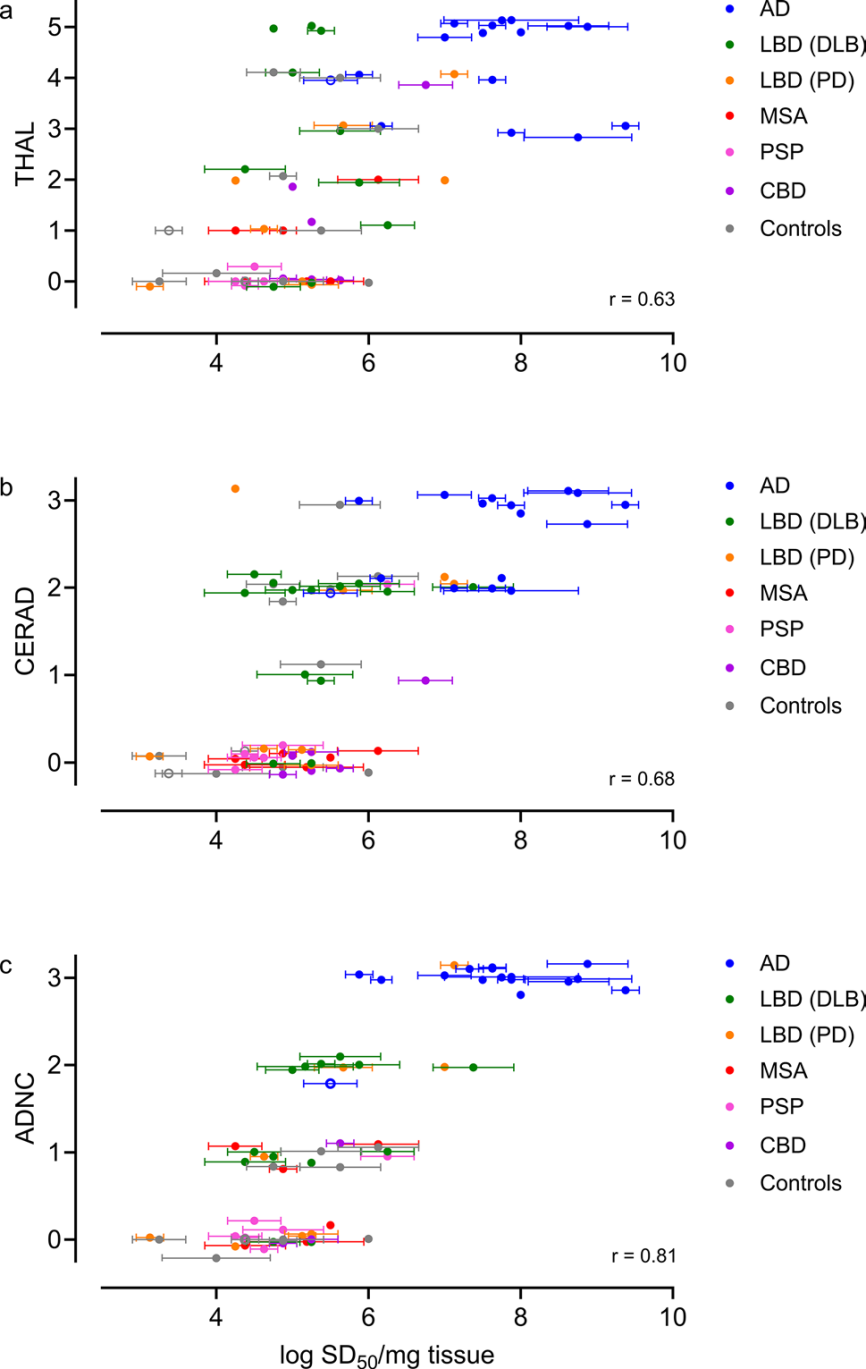
3R/4R tau seeding activities correspond with amyloid-β (A β) deposition and severity and overall AD neuropathologic change. 3R/4R tau seeding doses are shown as log SD_50_/mg brain tissue, with cases colored by primary diagnosis. Each data point represents an individual case, with 2–4 replicate determinations of seeding doses (mean ± standard deviation indicated). Seeding doses are shown as they correlate to **a** Thal phase of Aβ deposition (*r* = 0.63) [(AD (*n* = 16), LBD (DLB) (*n* = 10); LBD (PD) (*n* = 8); MSA (*n* = 6); CBD (*n* = 6); PSP (*n* = 5) and controls (*n* = 11)] **b** Consortium to Establish a Registry for Alzheimer’s Disease (CERAD) scores of neuritic amyloid plaques (*r* = 0.68) [(AD (*n* = 16), LBD (DLB) (*n* = 13); LBD (PD) (*n* = 8); MSA (*n* = 6); CBD (*n* = 6); PSP (*n* = 6) and controls (*n* = 12)] and **c** AD neuropathologic change (ADNC) (*r* = 0.81) [(AD (*n* = 16), LBD (DLB) (*n* = 13); LBD (PD) (*n* = 8); MSA (*n* = 6); CBD (*n* = 3); PSP (*n* = 6) and controls (*n* = 7)]

AD neuropathologic change (ADNC) is characterized by the accumulation of both Aβ plaques and tau NFTs in the brain. To assess if tau seeding activities correspond with severity of Aβ pathology, we evaluated the correlation of 3R/4R tau seeding activities with Thal phases [57] and Consortium to Establish a Registry for Alzheimer Disease (CERAD) scores [44] as indicators of Aβ deposition and the abundance of neuritic amyloid plaques, respectively. 3R/4R tau seeding activities showed modest correlations with Thal phase and CERAD scores (*r* = 0.63, *p* < 0.0001 and *r *= 0.68, *p* < 0.0001 respectively) (Fig. 4a, b). Overall ADNC scores showed comparatively better correlation with SD_50_ values (*r* = 0.81, *p* < 0.0001) (Fig. 4c).

### 3R/4R tau seeds detected prior to and across stages of AD neuropathology share qualitative properties

#### Early and late-stage 3R/4R tau seeds share properties with NFTs of sarkosyl-insolubility and protease resistance

**Fig. 5:**
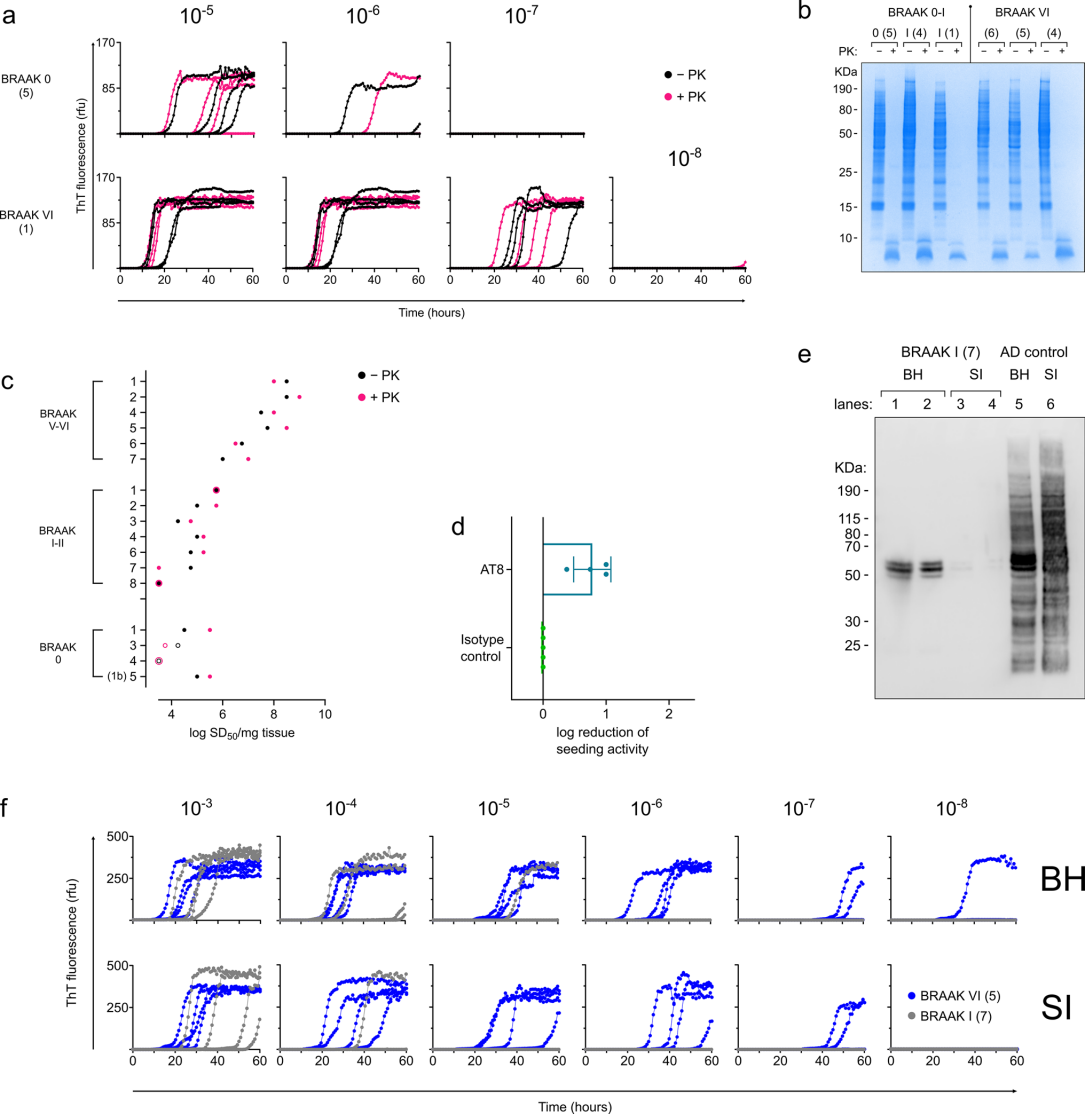
3R/4R tau seeds include hyperphosphorylated and sarkosyl-insoluble forms, with those that occur at early and late stages of AD neuropathology being largely protease resistant. **a** Brain homogenates from cases with absent/low Braak scores (0–II) or those designated AD (Braak V–VI) were treated with proteinase K (PK) prior to RT-QuIC analysis. Protease treated and mock-treated brain homogenates were used to seed the RT-QuIC assay. Endpoint-dilution analysis indicated quantitative seeding doses are comparable even after PK digestion. **b** SDS-PAGE analysis of treated (+ PK, pink) and untreated (−PK, black) brain homogenates confirmed efficiency of PK digestion. **c** Seeding doses (log SD_50_ per mg tissue) for (+ PK, pink) and untreated (−PK, black) brain homogenates are shown per case as indicated. **d** Immunoprecipitation using phospho-tau antibodies deplete up to 1 log of seeding activity. Log reduction of seeding activity is shown for phospho-tau antibodies, and when compared to seeding activities in brain homogenate supernatants subjected to isotype depletion. Each data point represents the log reduction detected in an individual AD case (Braak VI, *n* = 4). **e** Braak I cases contain sarkosyl-insoluble tau, albeit at much lower levels than that found in Braak VI cases. Lane 1, 1:2 dilution of 10% w/v Braak I brain homogenate. Lane 2, 1:4 dilution of 10% w/v Braak I brain homogenate. Lane 3, assuming 100% recovery of sarkosyl-insoluble product, ~ 8.2X 10% brain homogenate equivalent. Lane 4, assuming 100% recovery of sarkosyl-insoluble product, ~ 2.7X 10% brain homogenate equivalent. Lane 5, 1:2 dilution of 10% w/v Braak VI brain homogenate. Lane 6, assuming 100% recovery of sarkosyl-insoluble product, ~ 8.2X 10% brain homogenate equivalent. **f** Sarkosyl insoluble tau filaments have seeding activities that approach those detected in the total brain homogenate from which they were extracted. Seeding doses are shown for sarkosyl-insoluble extract equivalents, assuming 100% tau recovery from the brain tissue homogenates from which they were derived. Numbers in parentheses indicate case numbers as shown through panels (**a**–**f**)

Recent studies have suggested that tau seeds that occur at early versus late stages of AD pathology (Braak ≤ IV versus Braak V/VI) are marked by different post-translational modifications [60] and might be representative of distinct tau seed populations. As tau RT-QuIC identified widespread tau seed spread prior to or at earlier Braak stages than previously identified, we wanted to determine if the qualitative features of seeds that occur at early versus late Braak stages might differ as it relates to biochemical and/or structural properties. To do this, we conducted comparative analysis of seeds that occur in Braak stage 0 and Braak stage I versus late Braak stage VI cases (Fig. 5). First, we subjected the brain homogenates to proteinase K (PK) prior to analysis by RT-QuIC to determine the extent to which the seeding arose from protease resistant, and potentially insoluble tau conformers (Fig. 5a–c; Supplementary Fig. 3, online resource). Mass spectrometry analysis confirmed remaining tau fragments/peptides in select PK-treated AD-brain homogenates. Endpoint-dilution analysis showed that seeding doses were largely comparable between the PK treated and untreated samples suggesting seeds that occur at Braak stage 0, I, and VI share characteristics of protease resistance (Fig. 5a–c; Supplementary Fig. 3, online resource), a feature most often suggested to be associated with more mature NFTs. NFTs are heavily phosphorylated, and we used phospho-tau antibodies for immunodepletion of tau seeds from AD cases (Braak VI, *n* = 4). On average, AT8 immunodepletion resulted in a 0.78 log reduction seeding activities from AD-brain homogenates, equivalent to an 83% reduction (Fig. 5d). NFTs and tau filaments that occur in AD are sarkosyl insoluble. We performed a sarkosyl extraction on mid-frontal tissue from Braak I and V/VI cases. Immunoblot indicated that we can detect some insoluble tau in Braak I cases, although at much lower levels than the sarkosyl-insoluble tau derived from AD cases (Fig. 5e & Supplementary Fig. 4, online resource). We assessed seeding activities of sarkosyl-insoluble extracts from Braak I and VI as they compared to the seeding activities of the brain homogenate from which they were derived. Sarkosyl insoluble tau equivalents (see “Methods” for details) from early (Braak I) and late (Braak VI) cases have seeding activities that approach tau seeding activities detected in their corresponding brain homogenate (Fig. 5f).

### Prevalence of co-occurring α-syn, 4R and 3R/4R seeds in different neurodegenerative diseases

#### Tau seed isoform is selectively indicated and reproducibly quantifiable using different recombinant protein substrates with three independent tau RT-QuIC assays

**Fig. 6:**
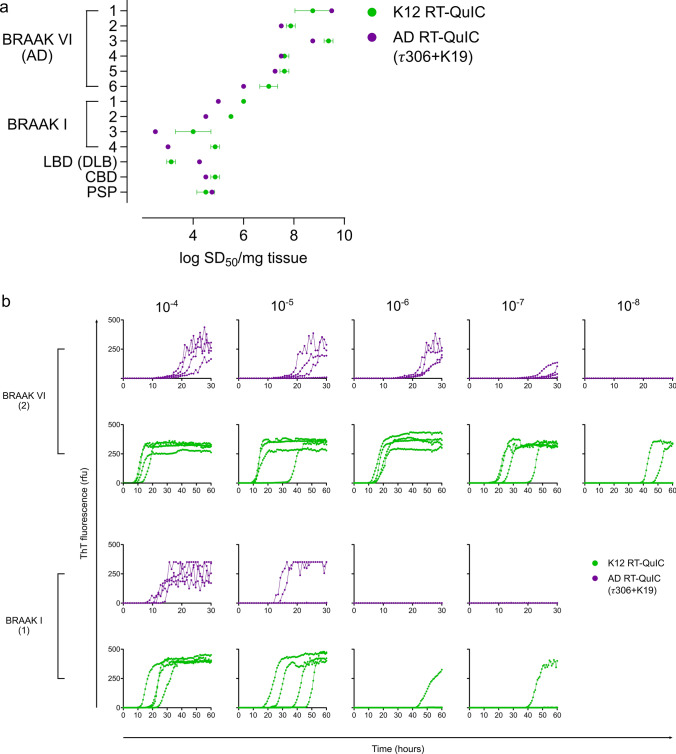
3R/4R tau seeds share qualitative characteristics in seeding efficiency with two independent types of 3R/4R tau selective RT-QuIC assays. **a** Seeding doses (shown as log SD_50_/mg brain tissue ± standard deviation) were determined for select Braak I and VI cases using both the K12 and AD RT-QuIC. **b** Endpoint-dilution analysis used to determine log SD_50_ values in **a** are shown for representative cases of Braak I and VI. Each curve represents an individual well, analyzed in quadruplicate for the tissue dilutions indicated. Bracketed numbers correspond to cases per Fig. 5c

**Fig. 7:**
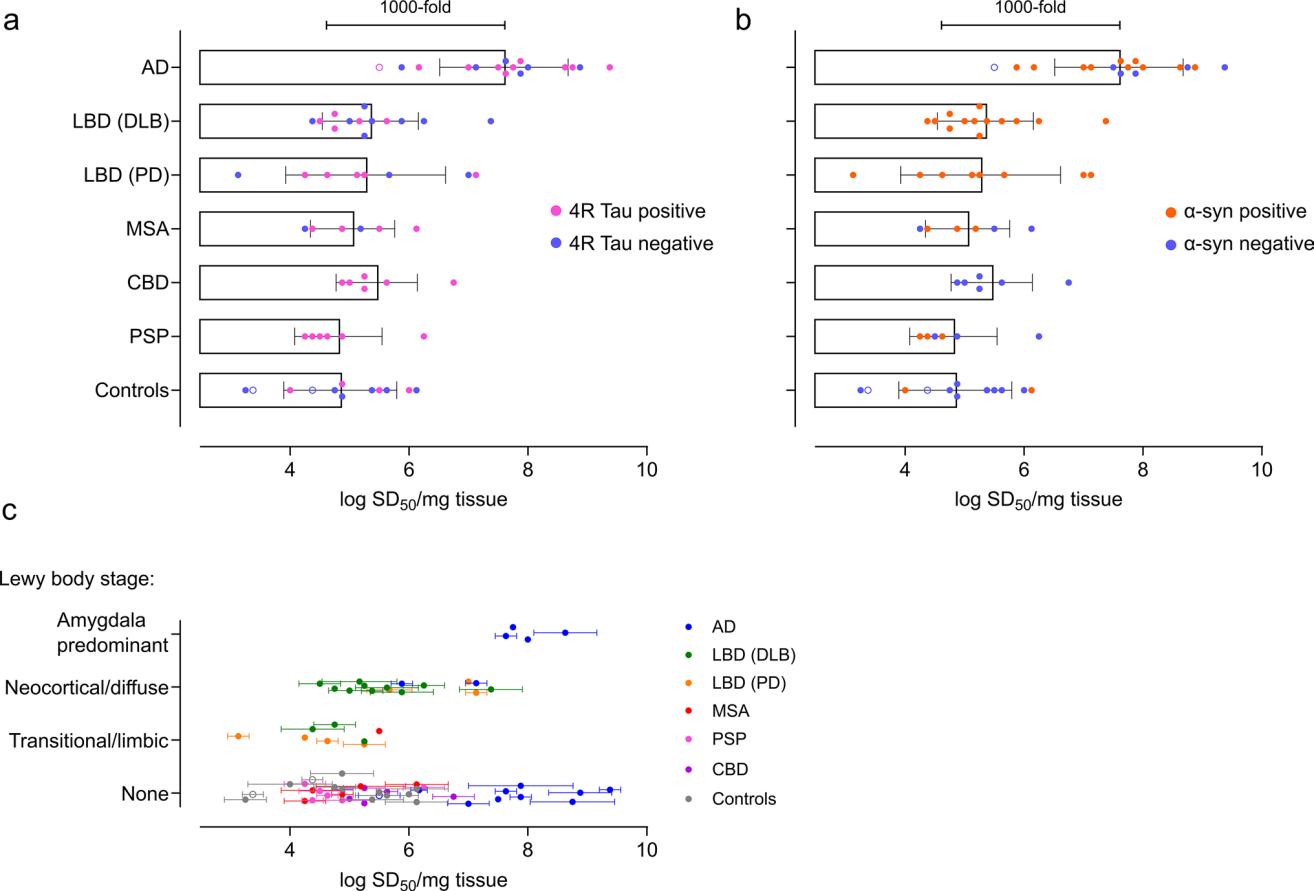
Prevalence of co-occurring 3R/4R, 4R tau and α-synuclein seeds. Binary analysis for co-occurring **a** 4R tau and **b** α-synuclein seeds were determined for all cases. 3R/4R tau seeding activities are as shown, with cases identified to also contain (positive or negative) 4R tau seeding activities colored in pink (**a**) and α-synuclein seeding activities in orange (**b**). AD (*n* = 16); LBD (DLB) (*n* = 13); LBD (PD) (*n* = 8); MSA (*n* = 6); CBD (*n* = 6); PSP (*n* = 6); controls (*n* = 12). **c** Higher tau seeding doses occur with neocortical and amygdala-predominant stages of Lewy body disease. 3R/4R tau seeding doses are shown as they correspond to Lewy body stage [40], with data points colored by primary diagnosis. Each data point represents the average seeding dose (*n* = 2–4) of an individual case. AD (*n* = 16); LBD (DLB) (*n* = 13); LBD (PD) (*n* = 8); MSA (*n* = 6); CBD (*n* = 6); PSP (*n* = 6); controls (*n* = 12)

We subsequently analyzed seeding activities using a second RT-QuIC assay that is selective for AD tau seeds [31] while using different recombinant substrates than those of the K12 RT-QuIC assay. Seeding activities were largely comparable between the two assay outputs suggesting quantitative differences assessed between Braak I and VI cases are not specific to the 3R/4R RT-QuIC assay used (Fig. 6). We additionally used 4R RT-QuIC to confirm tau seed isoform, and to evaluate 4R tau seed occurrence in 4R tauopathy and across non-4R tauopathy cases (Fig. 7a). Endpoint-dilution analysis to quantitate 4R tau seeds indicates 10^5^–10^7^ seeding doses in progressive supranuclear palsy (PSP) cases as a 4R tauopathy, and some 4R tau seeding in select Alzheimer’s cases albeit at multi-log lower levels when compared to 3R/4R tau seeding levels (Supplementary Fig. 5, online resource). This further confirms selectivity of tau RT-QuIC assays as it relates to specific tau seed detection and as has previously been indicated [11, 31, 43]. 4R seeds were absent from most of the normal cases, indicating tau seeding activities arise from 3R/4R tau in pathologically normal brains.

#### α-syn and tau seeds commonly co-occur

The cases evaluated for 3R/4R tau seeding activities include a number of primary synucleinopathies, including LBD (DLB) (*n* = 13), LBD (PD) (*n* = 8), and MSA (*n* = 6). To evaluate for co-occurring α-syn seeds in AD or other tauopathies, we used α-syn RT-QuIC to screen cases for α-syn seeding activity (Fig. 7b). α-syn seeds were detectable in 100% of the LBD cases analyzed, and in 50% of the MSA cases. α-syn seeds were also noted in 63% of the AD and 50% of the PSP cases (Fig. 7b). No α-syn seeds were detected in the CBD cases (*n* = 6) analyzed in this study. Consistent with prior reports that tau and α-syn aggregates can be frequently identified histologically as co-pathologies in brain tissue [48, 50] this suggests that 3R/4R tau and α-syn seeds also commonly co-occur across a spectrum of neurodegenerative diseases.

The co-occurrence of α-syn aggregates has been reported to synergistically accelerate tau accumulation, compared to when tau aggregates occur alone [18, 23, 62]. However, previous reports of these synergistic effects have largely relied on histological or fluorescent readouts of accumulating aggregates. We assessed frontal lobe tau seeding doses in cases representative of different Lewy body stages [40] (Fig. 7c). Our data indicated frontal cortex tau seeding doses are higher in cases with neocortical and amygdala Lewy body pathology (Fig. 7c), consistent with known pathologic correlations. Further study is required to determine prevalence of tau seeds in more brainstem-centric LBD. While our data overall suggests that significant tau seeding activities occur in the presence or absence of α-syn seeds, our evaluations lack spatial information as it would occur in tissue and cannot address complex questions of if α-syn presence influences tau accumulation over time. However, importantly, this identifies that 3R/4R tau and α-syn seed co-occurrence is highly prevalent in synucleinopathies.

#### Braak stage, 3R/4R tau seeding doses, and sex are significantly related

**Fig. 8:**
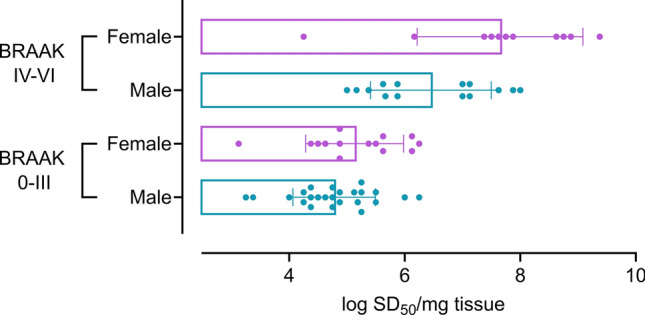
Tau seeding doses differ by sex at higher Braak stages. Tau seeding doses (SD_50_) are shown for cases with ≥ Braak IV or ≤ Braak III as it occurs by sex (Braak 0–III *M* = 24 *F* = 14; Braak IV–VI *M* = 14, *F* = 11). Each data point represents the average SD_50_ of an individual case. Tau seeding doses are higher in female cases with Braak staging ≥ IV when compared to males (*p* = 0.028)

Tau-PET scans of MCI and AD cases have indicated that in addition to having a higher prevalence of disease, females have more tau burden than males [55]. To investigate the relationship between sex and tau seeding doses, we used median quantile regression controlling for binary age (≤ 70 or ≥ 70), Braak stage (0–III, IV–VI) and the interaction between binary Braak stage and sex. We grouped 3R/4R tau seeding doses analyzed across all cases by sex (Fig. 8) and by Braak ≤ III (absent & low/intermediate AD tau neuropathology) or Braak ≥ IV (high AD tau neuropathology). Braak stage is significantly related to tau seeding doses (*p* = 0.003), and the interaction term between Braak stage and sex is significant (*p* = 0.028). Median tau seeding doses are 1.625 logs higher in ≥ Braak IV female cases when compared to males. Female cases of Braak stage IV–VI have a 2.875 log higher median tau seeding doses and males cases a higher median of 1.375 logs when compared to the sex matched Braak 0–III group. This suggests that, at least in the cases analyzed here, higher 3R/4R tau seeding doses occur in female cases with high AD tau neuropathology and supports further analysis with much larger representative groups of distinct neurodegenerative disease at different Braak stages.

## Discussion

Identification of widespread 3R/4R tau seeding at the earliest stages of AD processes suggests that tau seeds far precede any identifiable AD neuropathologic change or NFT accumulation. From a clinical perspective, the occurrence of seeds so early, and prior to neuropathologic change raises questions as to the window within which targeting seed-competent tau might be most therapeutically beneficial, and if such early 3R/4R tau seeds have the pathogenic equivalence of those that occur with late-stage AD neuropathology. Regardless, our data indicates that RT-QuIC seeding activities, evaluated in the mid-frontal lobe, quantitatively predict overall Braak stage and AD neuropathologic change, especially at higher Braak tau stages. Importantly, our study quantitates seeds using end-point dilution analysis to estimate seeding doses, and as such does not rely on interpretation of the RT-QuIC curve readouts such as lag phase or area under the curve, measures that can be subject to reaction conditions and operational parameters, and not necessarily directly correlative with seed quantities (reviewed in [36]). This improved resolution could aid efforts to identify molecular features associated with distinct disease states using emerging omics technologies.

The cases analyzed here include neuropathological diagnoses representing AD, 4R tauopathies, and synucleinopathies. Our data indicates that 3R/4R tau seeds are a prevalent co-occurrence in all cases assessed, albeit one that can quantitatively differ. Of note, our sample cohorts included normal cases, with absent or low Braak levels, yet these samples still harbor 3R/4R tau seeds, suggesting 3R/4R tau seeds as a more common entity or co-pathology at least in the aged cases assessed here. How frequently tau seeds occur broadly in aged individuals and the age at which tau seeds can first be detected will require more analysis across increased case numbers. Interestingly, while our current data indicates that while some younger cases (< 45 years at death) are negative or nearly negative for tau seeding activity at least in brain tissue dilutions that can be directly analyzed via RT-QuIC assay, seeding activities were observed in select young cases (Fig. 2 and Supplementary Fig. 2, online resource), albeit at lower levels when compared to more aged cases. Not all protein seeds are equivalent in properties of pathogenicity and transmissibility in a host [8], and we continue to investigate qualitative features of 3R/4R tau seeds without apparent pathogenicity, and the age-dependence of tau seed accumulation. However, our results are consistent with a recent preprint [32] that reported detection of tau seeds in cognitively normal cases with use of biosensor cells and an additional prior step to immunoconcentrate tau seeds. Importantly, our assays can detect such seeds without methods to concentrate seeds, and outside of the entorhinal cortex in cases without evidence of tau accumulation and at the earliest stages of Braak change (Braak I/II). We cannot completely rule out that, with RT-QuIC assessment of frozen tissue contralateral to the histopathologically characterized fixed hemisphere, there may be low levels of tau aggregates that would be histologically identifiable in our frozen tissue sections. Recent studies have indicated that use of T231 and S396/404 antibodies may identify even earlier tau species, before pretangles, with histopathological evaluation compared to the antibodies we used in our study here for neuropathological characterization [2]. However, the observation that the seeding activities are consistently and broadly observed across all cases > 45 years evaluated here supports that tau seeds occur prominently and at high levels without any, or certainly not significant amounts, of histopathological tau. Our use of tau knock-out mouse brain tissue as a negative control rules out the potential that our widespread observations of nearly ubiquitous 3R/4R tau seeding might reflect false positivity (i.e. spontaneous fibril formation in the assay). Select human cases that lack seeding activity confirms the baseline established with mouse tau knock-out brain homogenate (Supplementary Fig. 2, online resource).

As it relates to synucleinopathy, the cases with Lewy-related pathology stage indicated to be neocortical or amygdala-predominant showed the highest tau seeding activities. Of note, collectively LBD cases (inclusive of cases at transitional, neocortical, or amygdala-predominant stage) showed the greatest quantitative variance in 3R/4R tau seeding activities with a mean log SD_50_/mg of 5.32 and a range of 4 logs (3.13–7.38), presumably due to the different tau seeding activities noted at each stage (Fig. 7). Tau seeding doses were highest in the amygdala-predominant cases (AD, *n* = 4). Amygdala predominant Lewy body stage has previously been associated with severe Braak NFT stage (V–VI) and AD neuropathology [13, 24, 49, 58]. Our data is consistent with these observations, indicating that AD with amygdala-predominant Lewy body stage also has higher 3R/4R tau seed load.

There is growing recognition that multiple misfolded proteins commonly co-occur, at least in the setting of neurodegenerative disease (reviewed in [48]). This study indicates that 3R/4R tau seeds occur broadly across neurodegenerative diseases, but also at lower levels in non-diseased controls, including those with no evidence of neuropathologic tau accumulation. When we consider co-occurring protein seeds broadly, and with respect to how this ultimately impacts neuropathologic and disease outcome, recent attention has been paid to the consideration that co-occurring misfolded proteins may impact the rate and/or ability of individual seed-competent proteins to spread and propagate. While we focus on the selective quantitation of 3R/4R tau seeds in this study, we also assessed all cases for the co-occurrence of 4R tau and α-syn seeds. α-syn presence or absence did not determine the occurrence of tau seeding doses, at least those assessed in frontal cortex. This does not rule out an impact of α-syn and tau seed co-occurrence on disease duration and/or progression, or the possibility that co-occurring α-syn seeds might have impact on tau seed accumulation in a brain region-dependent way. Our data indicated that 3R/4R and 4R tau seeds also co-occur. 4R tau seeds were characteristic of 4R tauopathies, with occurrence noted in select other cases across neurodegenerative diseases although with a lower prevalence with comparison to 3R/4R tau seeds (Fig. 7 and Supplementary Fig. 5, online resource). Regardless, much more study is required to understand the importance of co-occurring protein seeds as it relates to neuropathological and clinical outcomes.

On a technical note related to co-occurring protein seeds, this should also include consideration if co-occurring proteins might otherwise influence assay readouts based on self-propagating properties such as the assays used here. Towards this, we have previously reported that inclusion of Aβ oligomers in sub or super stoichiometric quantities compared to tau within AD-brain homogenates did not significantly influence lag times noted for AD-brain seeded reactions [31]. In this study, we report that sarkosyl-insoluble tau has seeding activities that approach those of the brain homogenate from which they are derived. We note that some, but not all, sarkosyl-insoluble preparations also contained immunoreactive insoluble Aβ (data not shown). However, there were no systematic differences in normalized seeding activities of SI tau preparations with and without Aβ, suggesting Aβ presence per se, like we observed for α-syn co-occurrence, doesn’t significantly alter tau assay readouts. Limitations in our current study include the probability that the logarithmic working range of RT-QuIC assays, with use of tenfold dilutions here for seeding dose quantitation, may not resolve more subtle impacts of molecular interactions between co-occurring seeds as it relates to kinetics of fibril formation. In addition, assessments of seeding activities of brain homogenates removes the spatial aspects of seed distribution as it may occur in the brain. Assay conditions used here also likely do not fully replicate the structures of AD tau filaments as we know them to occur in brain [35], limiting their utility in addressing the complexities of how different protein seeds could impact formation and/or propagation of heterologous misfolded proteins and their structures that occur in vivo.

Our data suggests that tau seeds occur at higher levels in female cases of Braak stage ≥ IV when compared to males. Select diagnoses, including the LBD cases we analyzed here, were unequally distributed between males and females, and as such, we cannot assess if tau seeding activities differ by sex specifically in these groups. AD is more prevalent in females [39] with two-thirds of Americans diagnosed with AD being female [4]. Recent studies have begun to implicate specific molecular contributors to sex-dependent AD vulnerability [64], however, the mechanisms underlying this sex discrepancy are still not yet understood. Several reports have recently indicated that tau burden is higher, and more widespread in female mild cognitive impairment (MCI) patients than in males. An examination of tau-PET signal in 419 cases from four different cohorts found higher rates of tau accumulation in females and younger amyloid-β-positive individuals [55]. PET data from clinically normal and MCI subjects from the Alzheimer’s Disease Neuroimaging Initiative (ADNI) indicated women had a higher network density with higher levels of and more widespread tau burden [54]. Limitations of our study include the relatively small sample numbers, and as they occur over the different neurodegenerative diseases with known sex-specific prevalence differences (e.g. AD versus LBD). Without systematic regional testing, we also cannot rule out that sex-specific differences in tau seeding doses are restricted to the frontal cortex, and as it reflects disease-specific neuroanatomic distribution. Further studies are warranted to better understand at what pathologic stage tau seeding doses differ by sex and what implications this has for disease progression.

3R/4R tau seeding doses measured here are significantly related to Braak stage, an overall measure of NFT accumulation. Oligomeric versus fibrillar (e.g. NFTs) tau assemblies have been suggested as a more neurotoxic form of tau [33, 34]. Other studies have suggested that tau seed-competence is related to the accumulation of specific tau fragments and post-translational modifications [60] with AD disease processes. Our data indicates seeding activities are detectable far before histologically visible NFT deposits, but also that seeds share qualitative features at both early and late Braak stages (Fig. 5 and Supplementary Fig. 2, online resource), at least by measures of protease resistance, a characteristic most often associated with highly ordered and often amyloid structures. This suggests seeds, even at early stages of disease, may represent a pre-fibrillar or sub-fibrillar self-propagating species of comparable characteristics to the conformers that eventually make up large, histologically visible NFTs. Regardless, this study confirms prior findings [31, 43] indicating that 3R/4R tau seeding activities are orders of magnitude higher in AD brain compared to most other types of neurodegenerative diseases.

The mechanisms underpinning our observation that tau seeding activities increased most drastically with a multi-log jump in detectable seeding activities (Fig. 2) at Braak stage V and VI remain unclear. One possibility might be that at early stages of disease tau seeds can be maintained at lower levels, whereas at later stages of disease, and when clinical manifestations of AD occur, higher quantities of seeds accumulate. Alternatively, this may indicate that seeding activities in the mid-frontal lobe at < Braak IV represent qualitatively distinct tau seeds when compared to those of AD or seed-competent forms of tau that are part of a non-disease related process. A recent paper using mass spectrometry suggests an ordered occurrence of post-translational modifications (PTMs) on tau that coincides with tau aggregation processes and NFT formation, with a specific subset indicating a higher prevalence in AD [60]. Our data indicates that tau seeds from cases without Braak pathology and at early and late Braak stages share qualitative features, being largely protease resistant (akin to structured filaments) and able to seed misfolding of different protein substrates in independent 3R/4R tau RT-QuIC assays (Fig. 6). As the biggest noted difference in tau seeding activity between early and later Braak stages is quantitative, this may suggest that late-stage AD-related tau seeds occur early but as a small subpopulation detectable only using highly sensitive techniques such as RT-QuIC. Alternatively, early tau seeds, while sharing some qualitative properties with late-stage AD seeds, might represent biochemically and/or structurally distinct tau forms generated independently of pathogenic outcomes, and we cannot rule out the possibility that they occur regularly in normal individuals.

This study has several limitations: (1) Confirmation of the results indicated by the 3R/4R RT-QuIC assays with a second method was not possible at this time as current analogous and widely available HEK293T tau biosensor cell methodologies detection readouts using 3R&4R or 4R constructs do not necessarily differentiate between structurally distinct 3R/4R versus 4R tau seeds within a sample. Further, it has been reported that detection of seeds in non-AD cases using biosensor cells includes an added prior step of immunoprecipitation to concentrate seeds out of brain tissue using a proprietary antibody [25, 32]. By comparison, RT-QuIC analysis used here allows for seed selectivity to discriminate AD (i.e. 3R/4R) from 4R tau seeds, with multi-log specificities, and without a prior concentration step. However, future experiments to confirm our results here using other methods with comparable detection sensitivities and tau seed selectivity would be valuable towards further establishing quantitative parameters of tau seeds that occur at different stages of disease. (2) Our data here indicates AT8 phospho-tau antibodies can immunodeplete up to 1 log of seeding activities from AD cases. However, our study does not comprehensively evaluate additional aspects that might influence tau 3R/4R seed properties, such as the impact of PTMs like phosphorylation at early versus late Braak stages. Such future experiments to further explore tau PTMs as they relate to seed-competency and qualitative features of tau seeds at different disease stages could reveal critical aspects of the tau seeds that occur with stages of pathology and their relationship to disease processes.

Related to aspects of seed spread and accumulation, a recent study suggests that tau accumulation is governed by different rates before and after Braak III, such that after Braak III, the processes are rate-limited by local replication and not by spread from one region to the next [42]. Based on prior data, this study also suggests low levels of tau seeds are already present in neocortical regions at < Braak III. Our data supports this, but the sensitivity of RT-QuIC assay further suggests that even prior to Braak pathology the tau seed load is substantial at ~ 10,000–1,000,000 seeding doses per mg brain tissue. Further efforts, with increased numbers of cases, are required to determine why significant seed load is observed prior to Braak III, and mechanistically what this might mean when we consider the relationship of tau seeds with the rate of cognitive decline.

## Supplementary Information

Below is the link to the electronic supplementary material.

Supplementary file1 (PDF 4122 kb)

## References

[1] Aoyagi A, Condello C, Stohr J, Yue W, Rivera BM, Lee JC (2019). Abeta and tau prion-like activities decline with longevity in the Alzheimer's disease human brain. Sci Transl Med.

[2] Aragao Gomes L, Uytterhoeven V, Lopez-Sanmartin D, Tome SO, Tousseyn T, Vandenberghe R (2021). Maturation of neuronal AD-tau pathology involves site-specific phosphorylation of cytoplasmic and synaptic tau preceding conformational change and fibril formation. Acta Neuropathol.

[3] Arriagada PV, Growdon JH, Hedley-Whyte ET, Hyman BT (1992). Neurofibrillary tangles but not senile plaques parallel duration and severity of Alzheimer's disease. Neurology.

[4] Association As (2020). 2020 Alzheimer's disease facts and figures. Alzheimers Dement.

[5] Bankhead P, Loughrey MB, Fernandez JA, Dombrowski Y, McArt DG, Dunne PD (2017). QuPath: open source software for digital pathology image analysis. Sci Rep.

[6] Braak H, Braak E (1991). Neuropathological stageing of Alzheimer-related changes. Acta Neuropathol.

[7] Braak H, Alafuzoff I, Arzberger T, Kretzschmar H, Del Tredici K (2006). Staging of Alzheimer disease-associated neurofibrillary pathology using paraffin sections and immunocytochemistry. Acta Neuropathol.

[8] Caughey B, Kraus A (2019). Transmissibility versus pathogenicity of self-propagating protein aggregates. Viruses.

[9] Coughlin D, Xie SX, Liang M, Williams A, Peterson C, Weintraub D (2019). Cognitive and pathological influences of tau pathology in Lewy body disorders. Ann Neurol.

[10] Coughlin DG, Ittyerah R, Peterson C, Phillips JS, Miller S, Rascovsky K (2020). Hippocampal subfield pathologic burden in Lewy body diseases vs Alzheimer's disease. Neuropathol Appl Neurobiol.

[11] Coughlin DG, Goodwill VS, Standke HG, Kim Y, Coley N, Pizzo DP (2022). Selective tau seeding assays and isoform-specific antibodies define neuroanatomic distribution of progressive supranuclear palsy pathology arising in Alzheimer's disease. Acta Neuropathol.

[12] DeVos SL, Corjuc BT, Oakley DH, Nobuhara CK, Bannon RN, Chase A (2018). Synaptic tau seeding precedes tau pathology in human Alzheimer's disease brain. Front Neurosci.

[13] Dickson DW, Uchikado H, Fujishiro H, Tsuboi Y (2010). Evidence in favor of Braak staging of Parkinson's disease. Mov Disord.

[14] Dickson DW, Kouri N, Murray ME, Josephs KA (2011). Neuropathology of frontotemporal lobar degeneration-tau (FTLD-tau). J Mol Neurosci.

[15] Dougherty RM, Harris RJC (1964). Animal virus titration techniques. Techniques in experimental virology.

[16] Furman JL, Vaquer-Alicea J, White CL, Cairns NJ, Nelson PT, Diamond MI (2017). Widespread tau seeding activity at early Braak stages. Acta Neuropathol.

[17] Giannini LAA, Xie SX, McMillan CT, Liang M, Williams A, Jester C (2019). Divergent patterns of TDP-43 and tau pathologies in primary progressive aphasia. Ann Neurol.

[18] Giasson BI, Forman MS, Higuchi M, Golbe LI, Graves CL, Kotzbauer PT (2003). Initiation and synergistic fibrillization of tau and alpha-synuclein. Science.

[19] Gibbons GS, Banks RA, Kim B, Changolkar L, Riddle DM, Leight SN (2018). Detection of Alzheimer disease (AD)-specific tau pathology in AD and NonAD tauopathies by immunohistochemistry with novel conformation-selective tau antibodies. J Neuropathol Exp Neurol.

[20] Gibbons GS, Kim SJ, Robinson JL, Changolkar L, Irwin DJ, Shaw LM (2019). Detection of Alzheimer's disease (AD) specific tau pathology with conformation-selective anti-tau monoclonal antibody in co-morbid frontotemporal lobar degeneration-tau (FTLD-tau). Acta Neuropathol Commun.

[21] Greenberg SG, Davies P (1990). A preparation of Alzheimer paired helical filaments that displays distinct tau proteins by polyacrylamide gel electrophoresis. Proc Natl Acad Sci U S A.

[22] Groveman BR, Orru CD, Hughson AG, Raymond LD, Zanusso G, Ghetti B (2018). Rapid and ultra-sensitive quantitation of disease-associated alpha-synuclein seeds in brain and cerebrospinal fluid by alphaSyn RT-QuIC. Acta Neuropathol Commun.

[23] Guo JL, Covell DJ, Daniels JP, Iba M, Stieber A, Zhang B (2013). Distinct alpha-synuclein strains differentially promote tau inclusions in neurons. Cell.

[24] Hamilton RL (2000). Lewy bodies in Alzheimer's disease: a neuropathological review of 145 cases using alpha-synuclein immunohistochemistry. Brain Pathol.

[25] Hitt BD, Vaquer-Alicea J, Manon VA, Beaver JD, Kashmer OM, Garcia JN (2021). Ultrasensitive tau biosensor cells detect no seeding in Alzheimer's disease CSF. Acta Neuropathol Commun.

[26] Holmes BB, Furman JL, Mahan TE, Yamasaki TR, Mirbaha H, Eades WC (2014). Proteopathic tau seeding predicts tauopathy in vivo. Proc Natl Acad Sci U S A.

[27] Irwin DJ, Grossman M, Weintraub D, Hurtig HI, Duda JE, Xie SX (2017). Neuropathological and genetic correlates of survival and dementia onset in synucleinopathies: a retrospective analysis. Lancet Neurol.

[28] Kaufman SK, Del Tredici K, Thomas TL, Braak H, Diamond MI (2018). Tau seeding activity begins in the transentorhinal/entorhinal regions and anticipates phospho-tau pathology in Alzheimer's disease and PART. Acta Neuropathol.

[29] Kovacs GG, Robinson JL, Xie SX, Lee EB, Grossman M, Wolk DA (2017). Evaluating the patterns of aging-related tau astrogliopathy unravels novel insights into brain aging and neurodegenerative diseases. J Neuropathol Exp Neurol.

[30] Kovacs GG, Lukic MJ, Irwin DJ, Arzberger T, Respondek G, Lee EB (2020). Distribution patterns of tau pathology in progressive supranuclear palsy. Acta Neuropathol.

[31] Kraus A, Saijo E, Metrick MA, Newell K, Sigurdson CJ, Zanusso G (2019). Seeding selectivity and ultrasensitive detection of tau aggregate conformers of Alzheimer disease. Acta Neuropathol.

[32] LaCroix MS, Hitt BD, Beaver JD, Estill-Terpack S, Gleason K, Tamminga CA (2022). Tau seeding without tauopathy. Biorxiv.

[33] Lasagna-Reeves CA, Castillo-Carranza DL, Sengupta U, Clos AL, Jackson GR, Kayed R (2011). Tau oligomers impair memory and induce synaptic and mitochondrial dysfunction in wild-type mice. Mol Neurodegener.

[34] Lasagna-Reeves CA, Castillo-Carranza DL, Sengupta U, Guerrero-Munoz MJ, Kiritoshi T, Neugebauer V (2012). Alzheimer brain-derived tau oligomers propagate pathology from endogenous tau. Sci Rep.

[35] Lovestam S, Koh FA, van Knippenberg B, Kotecha A, Murzin AG, Goedert M (2022). Assembly of recombinant tau into filaments identical to those of Alzheimer's disease and chronic traumatic encephalopathy. Elife.

[36] Manca M, Kraus A (2020). Defining the Protein seeds of neurodegeneration using real-time quaking-induced conversion assays. Biomolecules.

[37] Mann DMA, Snowden JS (2017). Frontotemporal lobar degeneration: pathogenesis, pathology and pathways to phenotype. Brain Pathol.

[38] Masuda-Suzukake M, Nonaka T, Hosokawa M, Kubo M, Shimozawa A, Akiyama H (2014). Pathological alpha-synuclein propagates through neural networks. Acta Neuropathol Commun.

[39] Mazure CM, Swendsen J (2016). Sex differences in Alzheimer's disease and other dementias. Lancet Neurol.

[40] McKeith IG, Dickson DW, Lowe J, Emre M, O'Brien JT, Feldman H (2005). Diagnosis and management of dementia with Lewy bodies: third report of the DLB Consortium. Neurology.

[41] McKeith IG, Boeve BF, Dickson DW, Halliday G, Taylor JP, Weintraub D (2017). Diagnosis and management of dementia with Lewy bodies: fourth consensus report of the DLB Consortium. Neurology.

[42] Meisl G, Hidari E, Allinson K, Rittman T, DeVos SL, Sanchez JS (2021). In vivo rate-determining steps of tau seed accumulation in Alzheimer's disease. Sci Adv.

[43] Metrick MA, Ferreira NDC, Saijo E, Kraus A, Newell K, Zanusso G (2020). A single ultrasensitive assay for detection and discrimination of tau aggregates of Alzheimer and pick diseases. Acta Neuropathol Commun.

[44] Mirra SS, Heyman A, McKeel D, Sumi SM, Crain BJ, Brownlee LM (1991). The consortium to establish a registry for Alzheimer's disease (CERAD). Part II. standardization of the neuropathologic assessment of Alzheimer's disease. Neurology.

[45] Montine TJ, Phelps CH, Beach TG, Bigio EH, Cairns NJ, Dickson DW (2012). National institute on aging-Alzheimer's association guidelines for the neuropathologic assessment of Alzheimer's disease: a practical approach. Acta Neuropathol.

[46] Nelson PT, Dickson DW, Trojanowski JQ, Jack CR, Boyle PA, Arfanakis K (2019). Limbic-predominant age-related TDP-43 encephalopathy (LATE): consensus working group report. Brain.

[47] Ossenkoppele R, Schonhaut DR, Scholl M, Lockhart SN, Ayakta N, Baker SL (2016). Tau PET patterns mirror clinical and neuroanatomical variability in Alzheimer's disease. Brain.

[48] Rahimi J, Kovacs GG (2014). Prevalence of mixed pathologies in the aging brain. Alzheimers Res Ther.

[49] Raunio A, Kaivola K, Tuimala J, Kero M, Oinas M, Polvikoski T (2019). Lewy-related pathology exhibits two anatomically and genetically distinct progression patterns: a population-based study of Finns aged 85. Acta Neuropathol.

[50] Robinson JL, Lee EB, Xie SX, Rennert L, Suh E, Bredenberg C (2018). Neurodegenerative disease concomitant proteinopathies are prevalent, age-related and APOE4-associated. Brain.

[51] Saijo E, Ghetti B, Zanusso G, Oblak A, Furman JL, Diamond MI (2017). Ultrasensitive and selective detection of 3-repeat tau seeding activity in Pick disease brain and cerebrospinal fluid. Acta Neuropathol.

[52] Saijo E, Metrick MA, Koga S, Parchi P, Litvan I, Spina S (2020). 4-Repeat tau seeds and templating subtypes as brain and CSF biomarkers of frontotemporal lobar degeneration. Acta Neuropathol.

[53] Shi Y, Zhang W, Yang Y, Murzin AG, Falcon B, Kotecha A (2021). Structure-based classification of tauopathies. Nature.

[54] Shokouhi S, Taylor WD, Albert K, Kang H, Newhouse PA, I Alzheimer's Disease Neuroimaging (2020). In vivo network models identify sex differences in the spread of tau pathology across the brain. Alzheimers Dement (Amst).

[55] Smith R, Strandberg O, Mattsson-Carlgren N, Leuzy A, Palmqvist S, Pontecorvo MJ (2020). The accumulation rate of tau aggregates is higher in females and younger amyloid-positive subjects. Brain.

[56] Studier FW (2005). Protein production by auto-induction in high density shaking cultures. Protein Expr Purif.

[57] Thal DR, Rub U, Orantes M, Braak H (2002). Phases of A beta-deposition in the human brain and its relevance for the development of AD. Neurology.

[58] Toledo JB, Gopal P, Raible K, Irwin DJ, Brettschneider J, Sedor S (2016). Pathological alpha-synuclein distribution in subjects with coincident Alzheimer's and Lewy body pathology. Acta Neuropathol.

[59] Wenning GK, Tison F, Ben Shlomo Y, Daniel SE, Quinn NP (1997). Multiple system atrophy: a review of 203 pathologically proven cases. Mov Disord.

[60] Wesseling H, Mair W, Kumar M, Schlaffner CN, Tang S, Beerepoot P (2020). Tau PTM profiles identify patient heterogeneity and stages of Alzheimer's disease. Cell.

[61] Wilham JM, Orru CD, Bessen RA, Atarashi R, Sano K, Race B (2010). Rapid end-point quantitation of prion seeding activity with sensitivity comparable to bioassays. PLoS Pathog.

[62] Williams T, Sorrentino Z, Weinrich M, Giasson BI, Chakrabarty P (2020). Differential cross-seeding properties of tau and alpha-synuclein in mouse models of tauopathy and synucleinopathy. Brain Commun.

[63] Woerman AL, Aoyagi A, Patel S, Kazmi SA, Lobach I, Grinberg LT (2016). Tau prions from Alzheimer's disease and chronic traumatic encephalopathy patients propagate in cultured cells. Proc Natl Acad Sci USA.

[64] Yan Y, Wang X, Chaput D, Shin MK, Koh Y, Gan L (2022). X-linked ubiquitin-specific peptidase 11 increases tauopathy vulnerability in women. Cell.

