# Supplementary material for: Tau seeds occur before earliest Alzheimer’s changes and are prevalent across neurodegenerative diseases
Source: Acta Neuropathol. 2023 May 8;146(1):31–50. doi: 10.1007/s00401-023-02574-0 (PMC10261243; doi:10.1007/s00401-023-02574-0)
Supplement: Supplementary file 1 — Supplementary file1 (PDF 4122 kb) [file 401_2023_2574_MOESM1_ESM.pdf]

Supplementary material for

Tau seeds occur before earliest Alzheimer's changes and are prevalent across neurodegenerative diseases

Matteo Manca[1*], Heidi G. Standke[1*], Danielle F. Browne[1], Mikayla L. Huntley[1], Olivia R. Thomas[1], Christina D. Orrú[2], Andrew G. Hughson[2], Yongya Kim[3], Jing Zhang[4], Curtis Tatsuoka[5], Xiongwei Zhu[1], Annie Hiniker[6], David G. Coughlin[3], Douglas Galasko[3], and Allison Kraus[1#]

[1]Department of Pathology, Case Western Reserve University School of Medicine, Cleveland, OH 44106

[2]Laboratory of Persistent Viral Diseases, Rocky Mountain Laboratories, National Institute of Allergy and Infectious Diseases, National Institutes of Health, Hamilton, MT 59840

[3]Department of Neurosciences, University of California San Diego, CA, 92093-0612

[4]Department of Population and Quantitative Health Sciences, Case Western Reserve University School of Medicine, Cleveland, OH, 44106

[5]Department of Medicine, University of Pittsburgh, Pittsburgh, PA 15232

[6]Department of Pathology, University of California San Diego, San Diego, CA, 92093-0612

*co-first authors

#correspondence to allison.kraus@case.edu

Correspondence:

Allison Kraus, PhD
Department of Pathology
Case Western Reserve University School of Medicine
2103 Cornell Road
Cleveland, OH 44106
Phone: 216-368-2422
Email: allison.kraus@case.edu

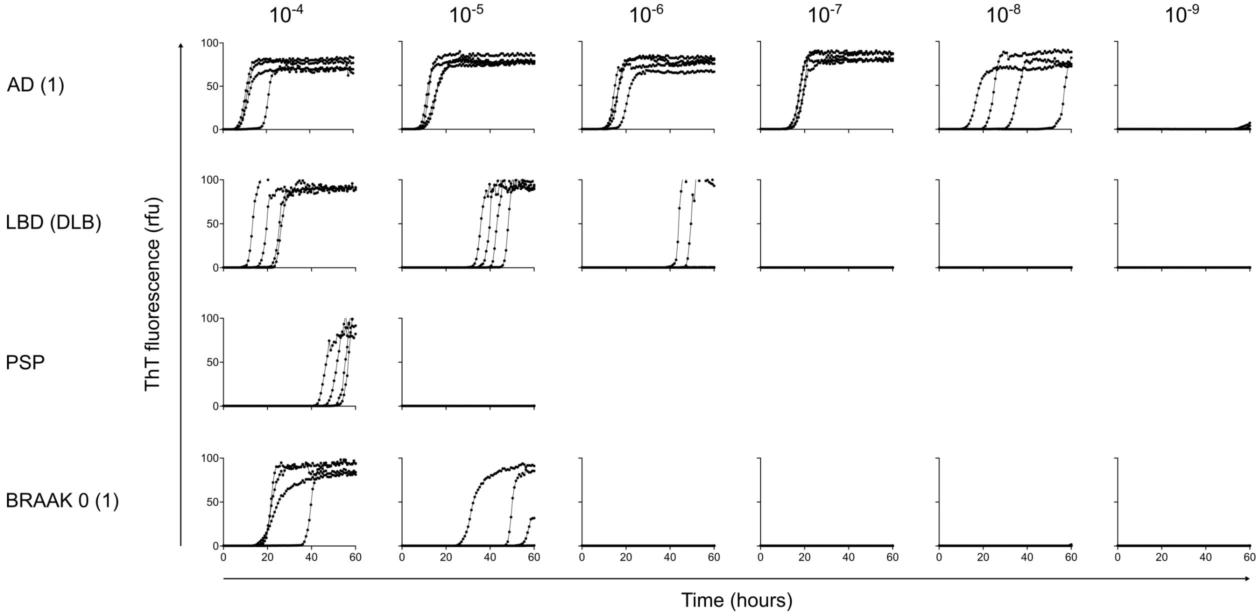

Supplementary Figure 1 Tau seeds occur prevalently across AD, primary synucleinopathies, 4R tauopathies, and controls. Endpoint dilution analysis is shown for representative AD, LBD (DLB), PSP, and control (Braak 0) cases. Each curve shows the relative ThT fluorescence of an individual well (normalized to baseline), with quadruplicate wells being analyzed at each brain tissue homogenate dilution. Endpoint dilution analysis was used to determine seeding doses, as shown in the main text. Parentheses indicate the case number as it corresponds to main text Fig. 5.

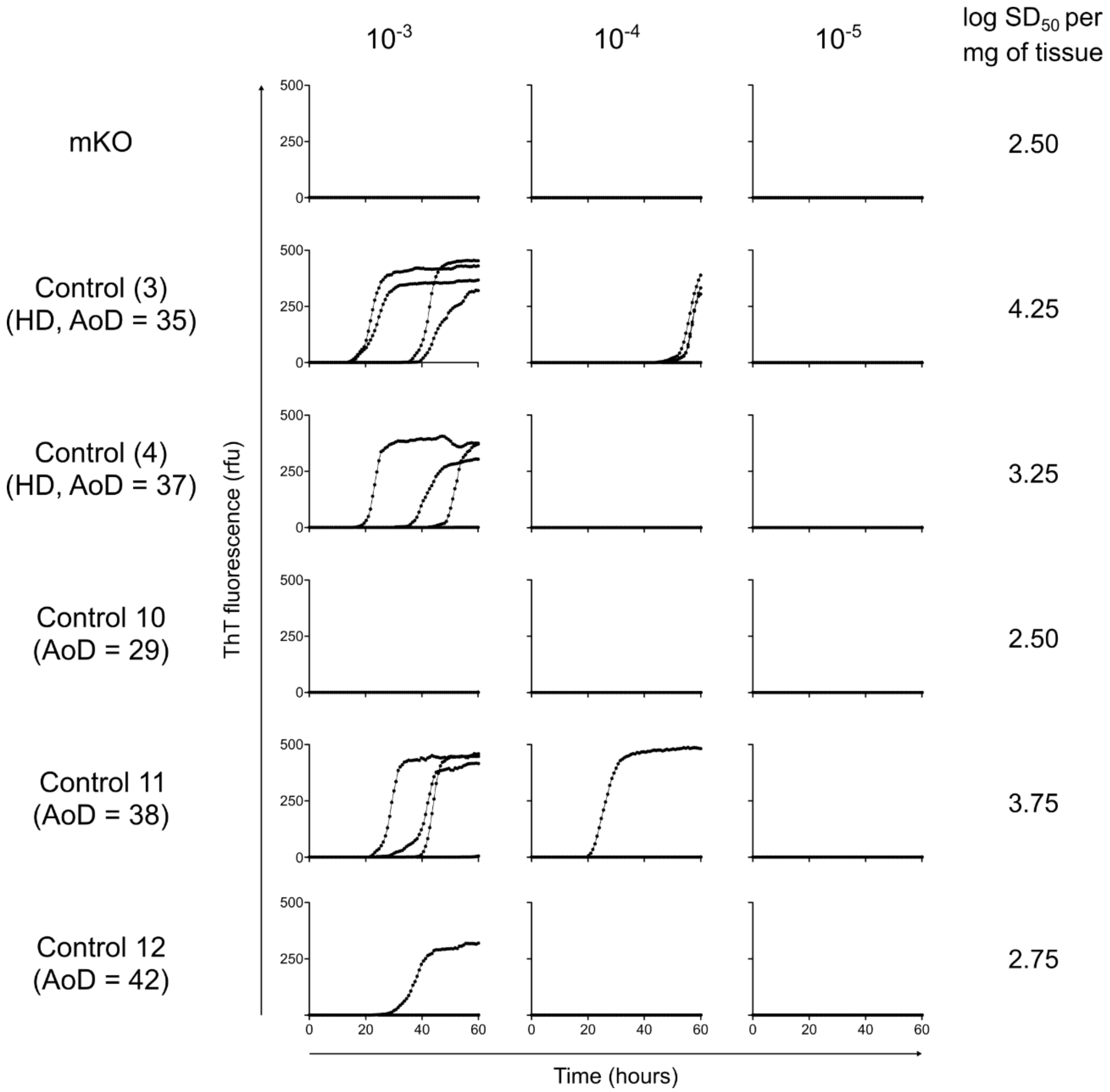

Case ID	Diagnosis	Age	Sex	PMI
Control (3)	Huntington's disease	35	F	14
Control (4)	Huntington's disease	37	M	NA
Control 10	No pathological diagnosis	29	M	18
Control 11	Glioblastoma multiforme	38	M	13
Control 12	Ischemia	42	M	2.5

Supplementary Figure 2 3R/4R tau seeding activities are detectable in some, but not all, normal cases <45 years. 3R/4R tau seeding activities were assessed in brain tissue from 5 cases <45 years. Controls 3 & 4 are also as described in Supplementary Table 1 and the main text. Parentheses indicate cases as they correspond to main text Fig. 5**c**. Each curve represents the relative ThT fluorescence values on an individual well of quadruplicate well analysis at the indicated brain tissue dilution (10^{-3} – 10^{-5}). Mouse tau knock-out (mKO) brain homogenate is also shown as a negative control. As described in methods, comparison of mKO and RT-QuIC negative human brain homogenate average baseline fluorescence reads validated the statistical comparability of baselines (t-test, p=0.35).

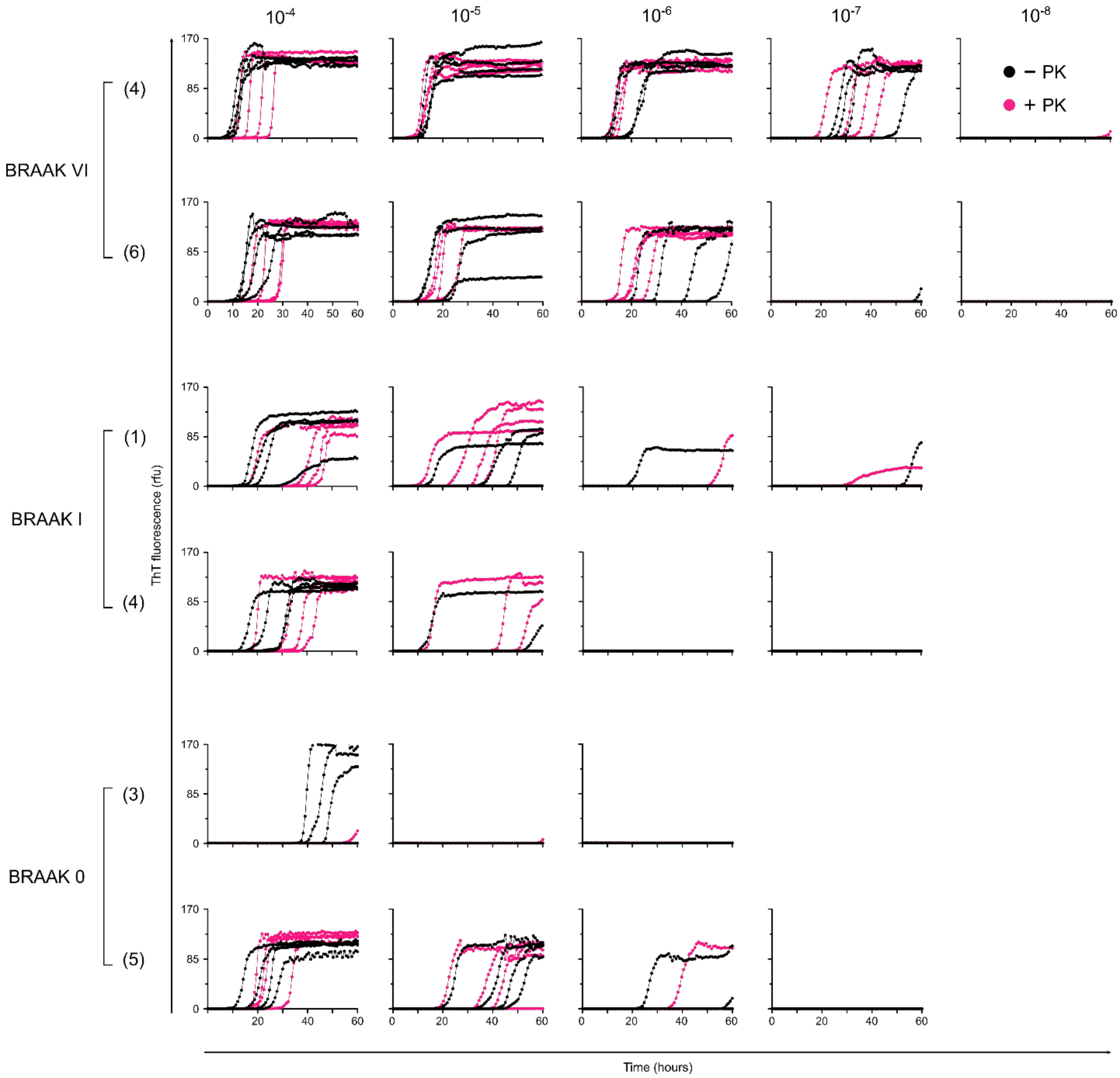

Supplementary Figure 3 3R/4R tau seeding activities are largely protease resistant. Brain tissue homogenates (BH) were subjected to proteinase K digestion (+PK, pink) and compared to tissue equivalents of untreated (-PK, black) samples with the 3R/4R tau RT-QuIC assay [1]. Endpoint dilution analysis is shown for the indicated AD (Braak VI) or control samples (Braak 0, I). Each curve represents the ThT fluorescence (relative fluorescence units, rfu) for an individual well, with quadruplicate wells seeded at each brain tissue dilution as indicated across the top. Endpoint dilution analysis was used to determine seeding doses as shown in the main text Fig. 5**c**, with the corresponding case numbers in parentheses as indicated beside the Braak stage.

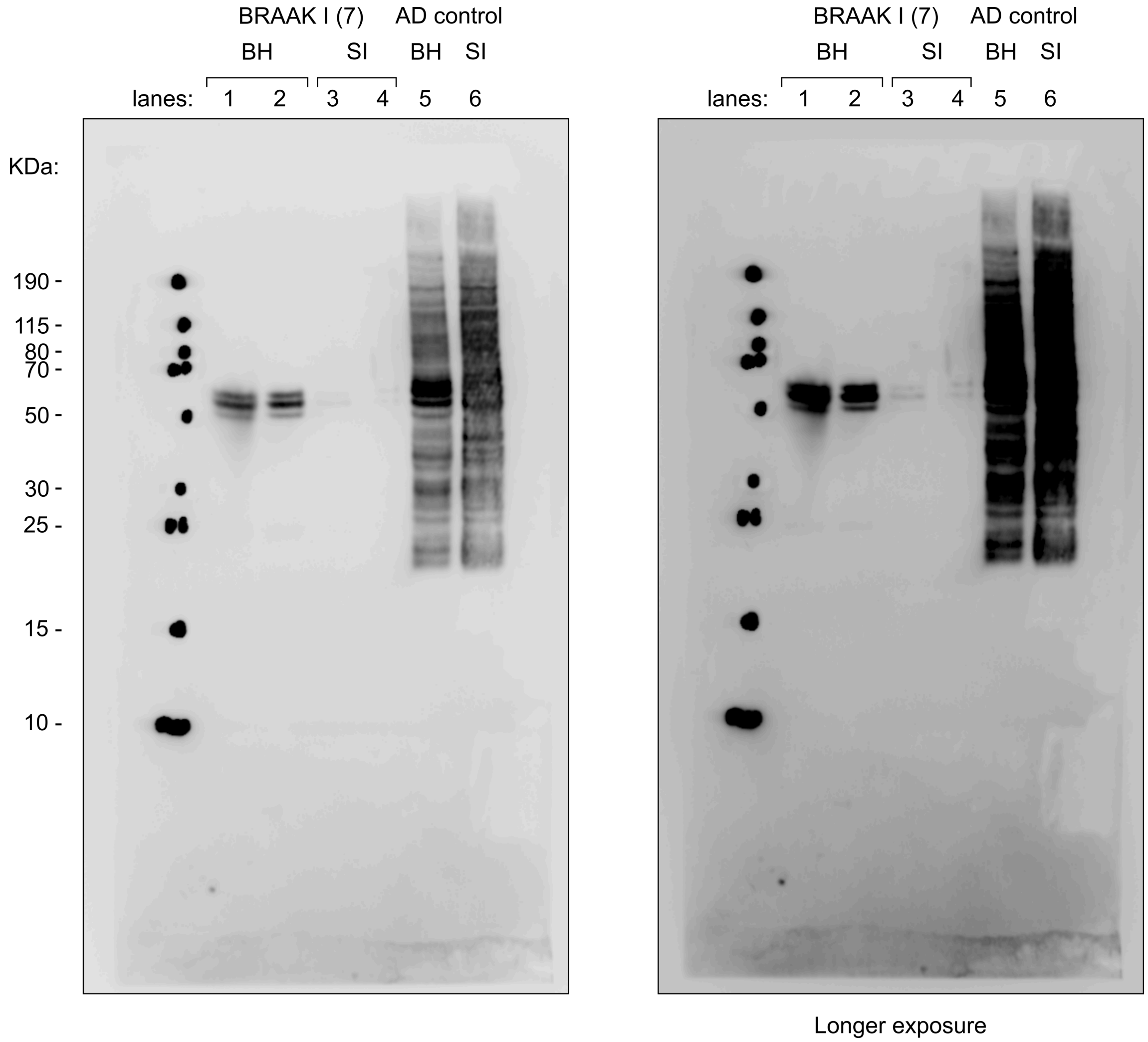

Supplementary Figure 4 Immunoblotting of sarkosyl insoluble tau in Braak I and AD cases. Immunoblot analysis using tau antibodies targeting the C-terminus indicate sarkosyl insoluble tau is detectable in frontal cortex tissue derived from Braak I and AD (Braak V/VI) cases. The blot on the right indicates a longer exposure. A cropped version of this blot is included in Fig. 5**e** of the main manuscript. Lane 1, 1:2 dilution of 10% w/v Braak I brain homogenate. Lane 2, 1:4 dilution of 10% w/v Braak I brain homogenate. Lane 3, assuming 100% recovery of sarkosyl insoluble product, ~8.2X 10% brain homogenate equivalent. Lane 4, assuming 100% recovery of sarkosyl insoluble product, ~2.7X 10% brain homogenate equivalent. Lane 5, 1:2 dilution of 10% w/v Braak VI brain homogenate. Lane 6, assuming 100% recovery of sarkosyl insoluble product, ~8.2X 10% brain homogenate equivalent. Numbers in parentheses correspond to cases as indicated in Fig. 5 of the main manuscript. BH, brain homogenate; SI, sarkosyl insoluble tau

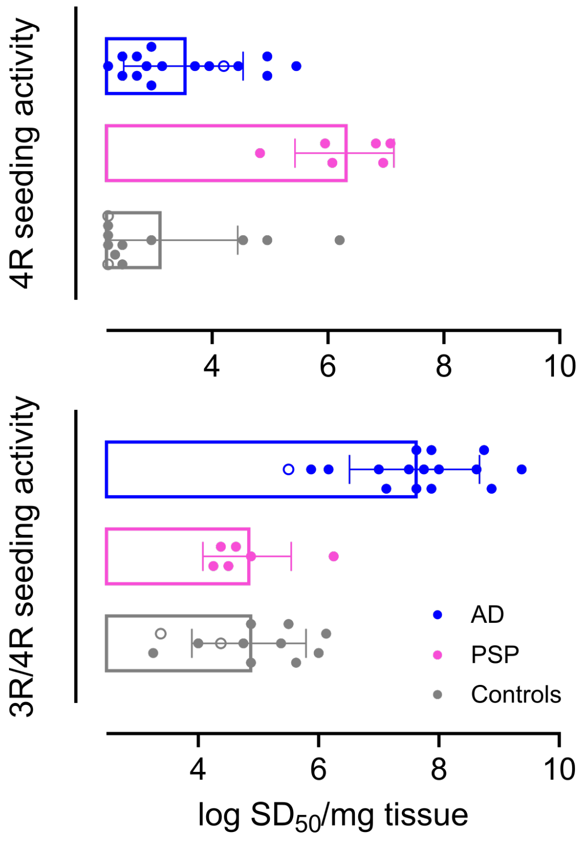

Supplementary Figure 5 3R/4R, but not 4R seeding activity is detected across most control cases. Seeding doses determined by 3R/4R or 4R RT-QuIC assay are shown for progressive supranuclear palsy (PSP), Alzheimer's disease (AD), and control cases. Each data point indicates the average SD_{50} determined for an individual case per mg of brain tissue. Open circles denote the intermediate AD case (blue) and the younger Huntington's cases (n=2, grey) (Supplementary Table 1).

Supplementary Table 1.

Pathological diagnosis	N	Clinical Diagnosis	Age at Death	Sex	Disease Duration	PMI (h)	Brain Weight (g)	Thal Phase	CERAD Stage	Braak Stage	ADNC	Lewy body stage
Young Controls: HD	2	HD=2	35,37	1 Male<br>1 Female	N=1<br>20	N=1<br>14	1049 (129)	A0=1<br>A1=1	C0=2	B0=2	Not=1<br>Low=1	None=0
Aged Controls: Normal or Mild AD changes	10	Normal=8<br>AD=2	N=9<br>81.1 (8.2)	5 Male<br>5 Female	N=2<br>10.5 (3.5)	N=6<br>9.7 (2.0)	N=9<br>1226 (117)	A0=4<br>A1=2<br>A2=1<br>A3=2	C0=4<br>C1=1<br>C2=4<br>C3=1	1b=1<br>I=8<br>II=1	Not=5<br>Low=4	None=10
AD	16	AD=15<br>Normal=1	79.8 (9.4)	7 Male<br>9 Female	N=12<br>10.8 (3.8)	N=14<br>10.6 (5.0)	N=15<br>1090 (191)	A2=4<br>A3=12	C2=6<br>C3=10	III=1<br>V=4<br>VI=11	Intermediate=1<br>High=15	None=10<br>Neocortical=2<br>Amygdala Predominant=4
LBD												
PD	8	PD=2<br>PDD=6	75.6 (6.1)	7 Male<br>1 Female	N=7<br>10.6 (5.7)	N=6<br>10.5 (7.1)	1278 (104)	A0=3<br>A1=3<br>A2=1<br>A3=1	C0=4<br>C2=3<br>C3=1	1a=1<br>I=3<br>II=1<br>IV=2<br>V=1	Not=4<br>Low=1<br>Intermediate=2<br>High=1	Limbic=5<br>Neocortical=3
DLB	13	AD=1<br>DLB=12	76.3 (9.8)	11 Male<br>2 Female	N=11<br>7.9 (3.7)	N=12<br>12.3 (6.9)	N=12<br>1379 (105)	A0=2<br>A1=3<br>A2=1<br>A3=7	C0=2<br>C1=2<br>C2=9	0=1<br>I=5<br>II=1<br>IV=6	Not=2<br>Low=5<br>Intermediate=6	Limbic=3<br>Neocortical=10
MSA	6	MSA-C=1<br>MSA-P=5	67.7 (9.5)	4 Male<br>2 Female	N=2<br>5, 15	N=5<br>14.4 (5.5)	1348 (210)	A0=3<br>A1=3	C0=6	1a=2<br>I=2<br>II=2	Not=3<br>Low=3	Not Applicable
CBD	6	AD=1<br>CBS=3<br>PPA=1<br>PSP-RS=1	74.0 (9.9)	2 Male<br>4 Female	N=5<br>5.8 (3.3)	N=5<br>10.2 (4.5)	1079 (119)	A0=3<br>A1=2<br>A3=1	C0=5<br>C1=1	I=1<br>II=1<br>Unstageable=4	Not=2<br>Low=1<br>Unstageable=3	None=6
PSP	6	PSP-F=1<br>PSP-P=1<br>PSP-RS=3<br>NA=1	N=5<br>72.8 (12.9)<br>NA=1	2 Male<br>4 Female	N=4<br>11.3 (3.9)	N=5<br>7 (5.2)	N=5<br>1104<br>(94.6)	A0=5<br>A2=1	C0=5<br>C1=1	I=4<br>II=1<br>IV=1	Not=5<br>Low=1	None=5

Abbreviations: PMI: post-mortem interval. AD: Alzheimer's disease, Vasc D: vascular dementia, LBD: Lewy body disease, PD: Parkinson's disease, DLB: dementia with Lewy bodies, MSA: multiple system atrophy, MSA-C: MSA cerebellar subtype, MSA-P: MSA parkinsonism subtype, CBD: corticobasal degeneration, CBS: corticobasal syndrome, PSP: Progressive supranuclear palsy, PSP-CBS: PSP corticobasal syndrome subtype, PSP-F: PSP frontal subtype, PSP-P: PSP parkinsonism subtype, PSP-RS: PSP Richardson syndrome, NA: not available. Thal phase, CERAD stage, Braak stage as per Braak et al. 2006 and 2011, and ADNC (AD neuropathological change) as per NIA-AA criteria Montine et al. 2012 and Lewy body stage as per McKeith et al 2017. All data displayed are means (standard deviation) and unless specified, represent the total cases in a category.

Supplementary Methods

UCSD QuPath digital image analysis parameters

Color deconvolution intensity thresholds were optimized per each staining run averaging values of red-blue-green color vectors and optimal minimal optical density values visually tuned from three to five representative slides per run using the Stain Vector Estimator tool in QuPath v0.2.0m2 as we have done previously [2-4].

	**Hematoxylin**			**DAB**			**DAB Threshold**
	Red	Green	Blue	Red	Green	Blue	
**AT8 Analysis**	0.738	0.621	0.263	0.344	0.533	0.783	0.156
**GT38 Analysis**	0.775	0.594	0.233	0.412	0.555	0.718	0.158

Supplementary Methods References

1. Metrick MA, 2nd, Ferreira NDC, Saijo E, et al. A single ultrasensitive assay for detection and discrimination of tau aggregates of Alzheimer and Pick diseases. *Acta Neuropathol Commun*. Feb 22 2020;8(1):22. doi:10.1186/s40478-020-0887-z
2. Coughlin DG, Ittyerah R, Peterson C, et al. Hippocampal subfield pathologic burden in Lewy body diseases vs. Alzheimer's disease. *Neuropathol Appl Neurobiol*. Dec 2020;46(7):707-721. doi:10.1111/nan.12659
3. Spotorno N, Coughlin DG, Olm CA, et al. Tau pathology associates with in vivo cortical thinning in Lewy body disorders. *Ann Clin Transl Neurol*. Dec 2020;7(12):2342-2355. doi:10.1002/acn3.51183
4. Giannini LAA, Xie SX, McMillan CT, et al. Divergent patterns of TDP-43 and tau pathologies in primary progressive aphasia. *Ann Neurol*. May 2019;85(5):630-643. doi:10.1002/ana.25465
